# Multi-omics analysis revealed the role of CYP1A2 in the induction of mechanical allodynia in type 1 diabetes

**DOI:** 10.3389/fgene.2023.1151340

**Published:** 2023-03-23

**Authors:** Hongjin Chen, Chenlong Liao, Xiaosheng Yang, Han Zhou, Yiwei Wu, Qiuyang Sun, Shuo Li, Wenchuan Zhang

**Affiliations:** Department of Neurosurgery, Ninth People’s Hospital Affiliated to Shanghai Jiao Tong University School of Medicine, Shanghai, China

**Keywords:** diabetic peripheral neuropathy, mechanical allodynia, lipid metabolism disorders, untargeted lipidomics, transcriptomics

## Abstract

**Background:** Mechanical allodynia (MA) is one of the leading clinical symptoms of painful diabetic peripheral neuropathy (PDPN), which is a primary reason for non-traumatic amputations, foot ulceration, and gait abnormalities in patients with diabetes. However, the pathogenic mechanisms of MA have not yet been fully elucidated, and there is no effective treatment. This study aims to study the potential pathogenetic mechanisms of MA and to provide targets for the therapy of MA.

**Methods:** A single intraperitoneal injection of streptozotocin induced type 1 diabetes in rat models. Subsequently, rats were divided into the control group, the diabetic group without MA, and the diabetic group with MA based on weekly behavioral assays. The differentially expressed lipids in the sciatic nerve of each group were detected using untargeted lipidomics, and the differentially expressed genes in the sciatic nerve of each group were detected by transcriptomics. The pathogenesis of MA was predicted using integrated analysis and validated by immunofluorescence staining and transmission electron microscopy.

**Results:** Untargeted lipidomics revealed the accumulation of a more severe lipid in MA rats. Transcriptomics results suggested that differentially expressed genes in MA rats were primarily related to lipid droplets and myelin sheath. Integrated analysis results indicated that the downregulation of Cytochrome P450 1A2 (CYP1A2) expression was closely linked to lipid metabolism disorders. Immunofluorescence staining demonstrated that down-regulation of CYP1A2 expression occurred in MA rats. Transmission electron microscopy results showed that more severe lipid droplet accumulation and myelin sheath degeneration occurred in MA rats.

**Conclusion:** Our findings imply that the downregulation of CYP1A2 expression leads to disorders of lipid metabolism and further leads to lipid droplet accumulation and myelin sheath degeneration, which might ultimately lead to the development of MA. Therefore, our study contributes to promoting the understanding of the molecular mechanisms of MA and providing potential targets for the clinical treatment of MA.

## Introduction

Diabetes mellitus (DM) remains a public health problem, affecting 422 million people worldwide (Hagedorn et al., 2022). DM and its complications are a major global health threat. Global diabetes-related health expenditures were estimated at 966 billion dollars in 2021 and are projected to reach 1,054 billion by 2045 (Sun et al., 2022). Additionally, more than 50% of individuals with DM develop neuropathy, the primary form of diabetic peripheral neuropathy (DPN) (Morgenstern et al., 2021). Painful diabetic peripheral neuropathy (PDPN) is the most challenging situation of DPN, which is a primary reason for non-traumatic amputations, foot ulceration, and gait abnormalities in patients with diabetes (Said, 2007). Notably, one of the significant clinical symptoms of PDPN is mechanical allodynia (MA). Despite a large number of individuals with diabetes and the severity of PDPN consequences, few studies have been conducted on PDPN, and the understanding of PDPN remains inadequate. It is still unclear why some patients with diabetes develop PDPN while others do not. To improve the quality of life of PDPN patients and to reduce the heavy economic burden caused by PDPN, more research on the pathogenesis of PDPN is urgently needed. A recent study suggests that dyslipidemia may also cause DPN in addition to hyperglycemia (Elafros et al., 2022). Thus, we hypothesized that lipid metabolism disorders correlate with MA in patients with diabetes. Considering the difficulty of obtaining clinical nerve samples and the long modeling period of type 2 diabetes, we used the streptozotocin (STZ)-induced type 1 diabetic rat animal model to conduct the study.

Lipidomics is a significant branch of metabolomics, focusing on the specific or exclusive study of lipids and lipid mixtures, including both targeted and untargeted lipidomics (Wishart, 2019). Importantly, lipidomic analysis is becoming a valuable tool in biomedical research. It can be used to discover biomarkers for the early diagnosis and prognosis of many diseases and to understand their biological functions (Yoon et al., 2022). As a result, understanding changes in lipid metabolism and their trafficking has mainly contributed to understanding the mechanisms behind various human diseases, including cancer, neurodegenerative diseases, diabetes, and obesity (Adibhatla and Hatcher, 2008; Lu et al., 2014). Transcriptomics is the complete set of transcripts in a cell, and their quantity, for a specific developmental stage or physiological condition. Understanding transcriptomics is essential for interpreting the genome’s functional elements, revealing the molecular constituents of cells and tissues, and understanding development and disease (Wang et al., 2009). Furthermore, multi-omics is a powerful and helpful approach to unraveling the molecular mechanisms of diseases (Xicota et al., 2019).

Cytochrome P450 1A2 (CYP1A2) encodes a member of the cytochrome P450 superfamily of enzymes. The cytochrome P450 proteins are monooxygenases that catalyze many reactions in drug metabolism and the synthesis of cholesterol, steroids, and other lipids. CYP1A2 is an anti-inflammatory CYP enzyme in humans. It primarily metabolizes arachidonic acid to distinct regioisomers of epoxyeicosatrienoic acids (EETs) to exert anti-inflammatory effects (Kwon et al., 2021). Notably, various drugs must be metabolized by CYP enzymes to exert their pain-relieving effects (Ingelman-Sundberg, 2004).

In the present study, we examined the differentially expressed metabolites and differentially expressed genes of rats in the control group (CON group), the diabetic group without MA (MA (−) group), and the diabetic group with MA (MA (+) group) by liquid chromatography-mass spectrometry (LC-MS) untargeted lipidomics and transcriptomics. We next found the regulatory network of differentially expressed genes and differentially expressed metabolites using multi-omics integrated analysis. Lipidomics and transcriptomics results were further validated by immunofluorescence staining and transmission electron microscopy. In conclusion, a more severe accumulation of lipid droplets and myelin sheath degeneration was found in the sciatic nerve of PDPN rats. Therefore, these results might be associated with the downregulation of CYP1A2 expression.

## Methods

### Animals

Sprague Dawley rats (200–240 g) were obtained from Shanghai Lab Animal Research Center. Rats were housed in specific pathogen-free conditions with free access to water and rat chow. All animal studies were approved by the Shanghai Jiao Tong University Animal Care and Use Committee and conducted by the Shanghai Jiao Tong University animal policies and guidelines established by the National Health and Family Planning Commission of China.

### Induction of type 1 diabetes

As previously reported (Liao et al., 2018), type 1 diabetes was induced by a single intraperitoneal injection of STZ (Solarbio, China) in rats. In brief, STZ was dissolved in citrate buffer (pH = 4.6) at a concentration of 1%. It was administered to the experimental group of SD rats *via* a single intraperitoneal injection at 60 mg/kg. The same volume of vehicle-injected control rats. Rats’ blood glucose levels were measured 3 days after injection using a blood glucose meter (Bayer Healthcare, United States ), and rats with a blood glucose level less than 11.1 mmol/L were injected intraperitoneally with STZ again. Body weight and blood glucose levels were measured weekly in all animals.

### Behavioral tests

As previously described, behavioral tests for MA were performed every week by von Frey filaments (North Coast, United States ) (Zhou et al., 2022). In brief, rats were separately placed in transparent plexiglass chambers on an elevated metal grid floor with 1 cm holes and probed from below. Subsequently, fibrils of various thicknesses (in this case, 1.4, 2, 4, 6, 8, 10, 15, and 26 g-force) were used to stimulate the hind paw of rats. The experiment begins by testing the response to the 10 g filament. Next, stimulation with thinner filaments was used if the rat exhibited a positive reaction, such as foot withdrawal. Conversely, if there was no positive response, the subsequent stimulation with thicker filaments was used instead. This continues until at least four readings are obtained after different reactions appear for the first time. If the force to be used is higher than 15 g or lower than 1.4 g, the threshold on this site is directly recorded as 15 g or 1.4 g, respectively (Deuis et al., 2017). The duration of each stimulation was 5 s, and the interstimulus interval was 15 s. The 50% paw withdrawal threshold (PWT) was determined by the up-down method of Chaplan et al. (Chaplan et al., 1994). On day 28 following STZ injection, diabetic rats were divided into the MA (+) group (the diabetic group with MA, PWT≤8 g) and the MA (−) group (the diabetic group without MA, PWT≥15) based on the results of the PWT.

### Extraction of the sciatic nerve in rats

Rats were anesthetized and secured in the supine position on a dissecting board, the chest cavity was opened to expose the heart, and then the pillow was inserted into the left ventricle of the heart, followed by clipping of the right auricle and infusion of 100 ml saline. Bilateral sciatic nerves were harvested from rats within 15 min after completion of perfusion, placed in liquid nitrogen for temporary storage, and later transferred to a negative 80 °C refrigerator for storage.

### LC/MS-based lipidomics

20 mg of tissue was added to 300 μl of water-methanol (V: V = 1:1, containing internal labels GCA-C13, CDCA-D4, CA-D4). Two tiny steel beads were added, placed at −20 °C for 2 min at a pre-chill temperature, and then added to the grinding machine (60 Hz, 2 min). Followed by adding 300 μL of chloroform, vortexing for 30 s, sonicating for 10 min, and leaving for 20 min at −20 °C. Subsequently, it was centrifuged for 10 min (13,000 rpm, 4°C), and 200 μL of the bottom layer of chloroform was loaded into the LC-MS injection vial. In the centrifuge tube, 300 μL of chloroform-methanol (V/V = 2:1, containing 0.1 mM BHT) was added to the sample, vortexing and stirring for 30 s sonicated for 10 min in a bathtub of ice water. Following evaporation, the lipid pellet in the LC-MS flask was again dissolved with 300 μl of isopropanol-methanol (V/V = 1:1), vortex for 30 s, sonication for 3 min, 20 μl of internal labels mixed isotope solution was added to the solution. The solution was transferred to a 1.5 ml centrifuge tube and allowed to stand for 2 h at −20 °C. The supernatant was centrifuged for 10 min (13,000 rpm, 4°C), and 150 μl of the supernatant was loaded into the flask along with the internal standard. A lining tube was used to load the supernatant into the LC-MS injection vial, which was then used for LC-MS analysis. Quality control (QC) samples were prepared by mixing equal volumes of extracts from the samples.

#### Chromatographic conditions

The column temperature was 55°C. Mobile phase A was acetonitrile/water (V/V = 60:40, containing 0.1% FA and 10 mM NH4COOH). Mobile phase B was acetonitrile/isopropanol (V/V = 10:90, containing 0.1% FA and 10 mM NH4COOH). The flow rate was 0.35 mL/min, and the injection volume was 5 ul.

#### Mass spectrometry conditions

The mass spectrometry system was a Qtrap 5,500 detection system (AB Sciex, United States ) with an electrospray ionization (ESI) source and an Analyst 1.7 workstation. Optimized conditions for mass spectrometry were gas curtain gas at 40 psi, ion spray voltage at 4,500/5500 V, source temperature at 400 °C, nebulizer gas at 50 psi, and auxiliary heater gas at 55 psi. Schedule-MRM mode was used for the high-throughput analysis of over 1,000 lipids.

The lipidomics analysis method was similar to the previously reported (Zhao et al., 2022). We first used unsupervised principal component analysis (PCA) to observe the overall distribution across samples as well as the stability of the entire analysis process (Supplementary Figure S2A), and then supervised partial least squares analysis (PLS-DA) and orthogonal partial least squares analysis (OPLS-DA) were used to distinguish the overall differences in metabolic profiles between groups and find the differential metabolites between groups (Supplementary Figures S2C-D). The *t*-test (Student’s t-test) and Fold change analysis were used to compare metabolites between the two groups. Additionally, volcano plots can be used to visualize *p* values and Fold change values, which is beneficial for screening differentially expressed metabolites. Hierarchical clustering was performed for all significantly differentially expressed metabolites to visually show the relationship between samples and differences in metabolite expression between samples. Lastly, pathway enrichment analysis was carried out for differentially expressed metabolites based on the Kyoto Encyclopedia of Genes and Genomes (KEGG) database.

### Transcriptomics analysis

Genes were first filtered based on the count mean value, and only genes with a mean count value greater than two were retained for the subsequent analysis. The counts of each sample gene were normalized using DESeq2 software (BaseMean value was used to estimate the expression), the difference fold was calculated, and the NB (negative binomial distribution test) was used to test the significance of the difference. Finally, the different protein-coding genes were screened based on the different fold and significance test results. The default conditions for screening differences were q < 0.05 and foldChange >2. The volcano plot was used to understand the overall distribution of differentially expressed genes and to cluster the differential expression of each group. After the differentially expressed genes were obtained, Gene Ontology (GO) enrichment analysis was performed on to describe their functions. Furthermore, pathway analysis of differentially expressed genes was performed using the KEGG database. Sequencing data was conserved in the National Center for Biotechnology Information (NCBI) database with the Gene Expression Omnibus (GEO) accession number GSE226315.

### Integrated analysis of lipidomics and transcriptomics

The Pearson correlation calculation method was used to calculate the correlation between differentially expressed genes and differentially expressed metabolites. Correlation heat maps were drawn based on the differentially expressed genes and metabolites association analysis results. Additionally, association network maps were drawn based on the differentially expressed genes and metabolites association analysis (*p* < 0.05). KEGG Markup Language (KGML) is one of the KEGG database sublibraries that contains both the relationships of graphical objects in the KEGG pathway and the information on immediate homologous genes in the KEGG GENES database. This information allows the network relationships of genes and metabolites to be obtained, facilitating a more systematic study of the interactions between the transcriptome and the metabolite.

### Immunofluorescence staining (IF)

Briefly, rats were anesthetized and then perfused with 100 ml of saline solution and 100 ml of 4% paraformaldehyde. Within 15 min after completion of perfusion, the bilateral sciatic nerves were removed and fixed overnight in 4% paraformaldehyde at 4 °C. Subsequently, the bilateral sciatic nerves were transferred into a 30% sucrose solution for at least 1 week. Tissue embedded into the Tissue-Tek OCT compound (Sakura Finetek) was frozen-sectioned. Next, the sciatic nerve sections were stained using anti-CYP1A2 (5 μg/ml, Abcam, ab22717) and anti-S100β(dilution 1:1,000, Abcam, ab52642). Furthermore, ImageJ measured the fluorescence intensity of immunostaining.

### Transmission electron microscope scan (TEM)

For transmission electron microscopy, rats were perfused transcardially with physiological saline and then with fixatives (2.5% glutaraldehyde, 2% paraformaldehyde) before dissecting sciatic nerves. The sciatic nerves were dehydrated with acetone and then embedded in beam capsules for ultrathin sectioning (50 nm). Furthermore, the grids were stained with uranyl acetate for 15 min, followed by lead citrate for 1 min. Images were captured under the electron microscope following standard protocols.

### Statistical analysis

The sample sizes were selected based on statistical requirements and the accepted norms of similar studies. The data are shown as mean ± SD. All statistical analyses were performed in GraphPad Prism (9.5.0), and R. Transcriptomics analysis and graph generation were performed in R (version 3.5.1). DESeq2 (1.22.2) was used for DEGs analysis. Clusterprofile (4.6.0) was used for enrichment analysis. Ggplots (3.3.6) was used for plotting. Lipidomics analysis and graph generation were performed in R (version 3.6.2). Ropls (1.22.0) was used for multivariate statistical analysis. Stats (4.0.5) was used for univariate statistics. Pheatmap (1.0.12), ggplot (3.3.6), ggrepel (0.9.1), and dplyr (1.0.9) were used for plotting. Integrated analysis and graph generation were performed in R (version 4.2.0). Pheatmap (1.0.12), ggplot (3.3.6), ggrepel (0.9.1), and dplyr (1.0.9) were used for plotting. *p* > 0.05 is not considered statistically significant, ∗ indicating *p* < 0.05, ∗∗ *p* < 0.01, and ∗∗∗ *p* < 0.001.

## Result

### Some STZ-induced diabetic rats developed MA

**FIGURE 1:**
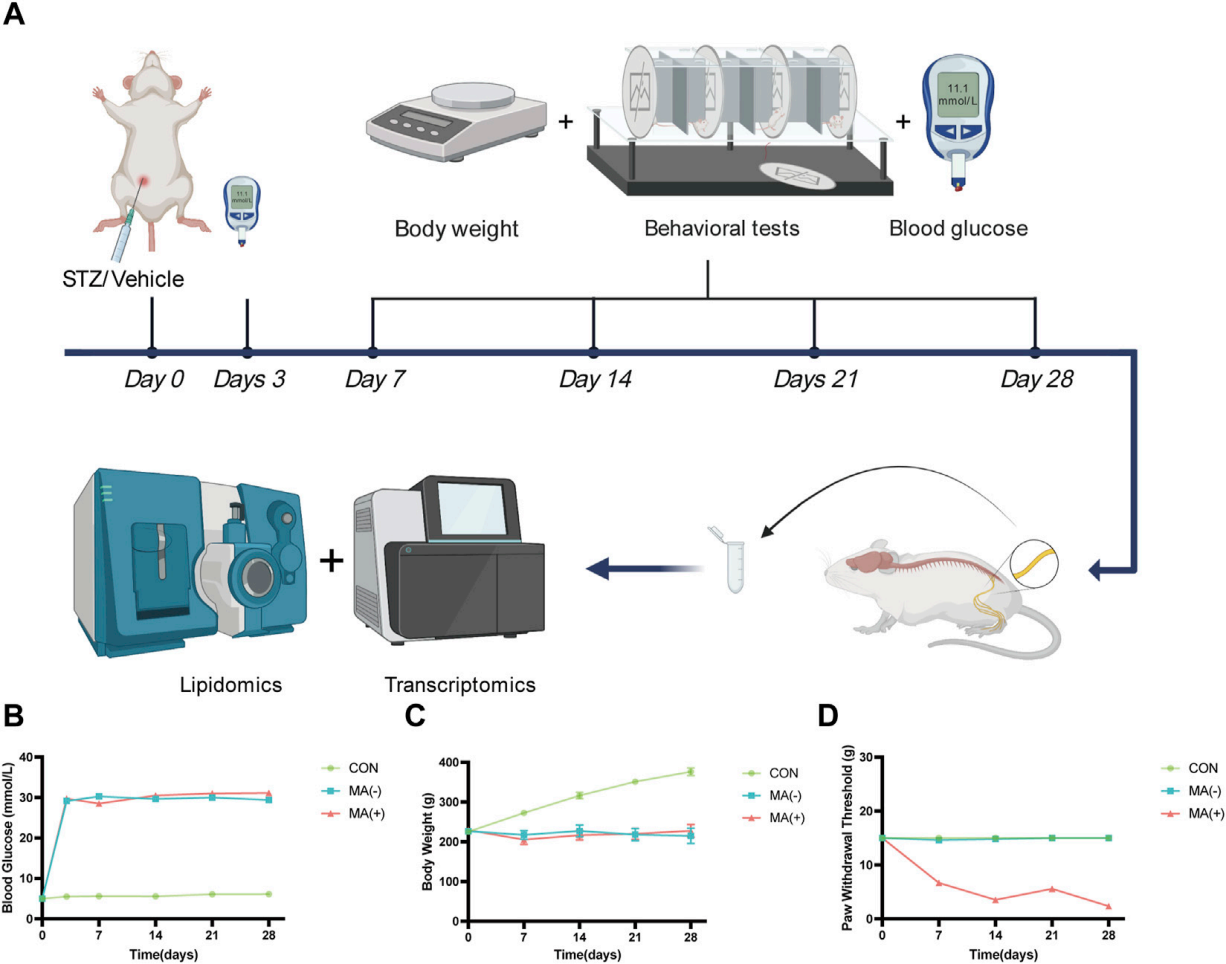
STZ-induced diabetic rats exhibit different pain phenotypes. **(A)**. Workflow of lipidomics and transcriptomics assay in STZ-inducted diabetic rats. **(B)**. The blood glucose levels of rats in the MA (+) and MA (−) groups were higher than those in the control group. **(C)**. The body weight levels of rats in the MA (+) and MA (−) groups were lower than those in the control group. **(D)**. The pain threshold in the MA (+) group was lower than in the MA (−) and control groups. Six rats in each group.

To understand the changes in the rats following STZ induction, body weight, blood glucose, and behavioral testing was performed on rats (Figure 1A). Compared with the control group, the level of blood glucose was found to be dramatically increased in the MA (−) group and MA (+) group (Figure 1B). In contrast, the level of body weight was found to be decreased in MA (−) group and MA (+) group (Figure 1C) after STZ intraperitoneal injection. In addition, rats in the MA (+) group showed a significant decrease in PWT level compared to the control group, whereas the PWT level of rats in the MA (−) group did not change significantly (Figure 1D). Therefore, these results suggested that rats in both the MA (+) and MA (−) groups had high blood glucose levels and weight loss. However, only rats in the MA (+) group showed a reduction in pain thresholds following the induction of STZ.

### More severe lipid accumulation was detected in the MA (+) group

**FIGURE 2:**
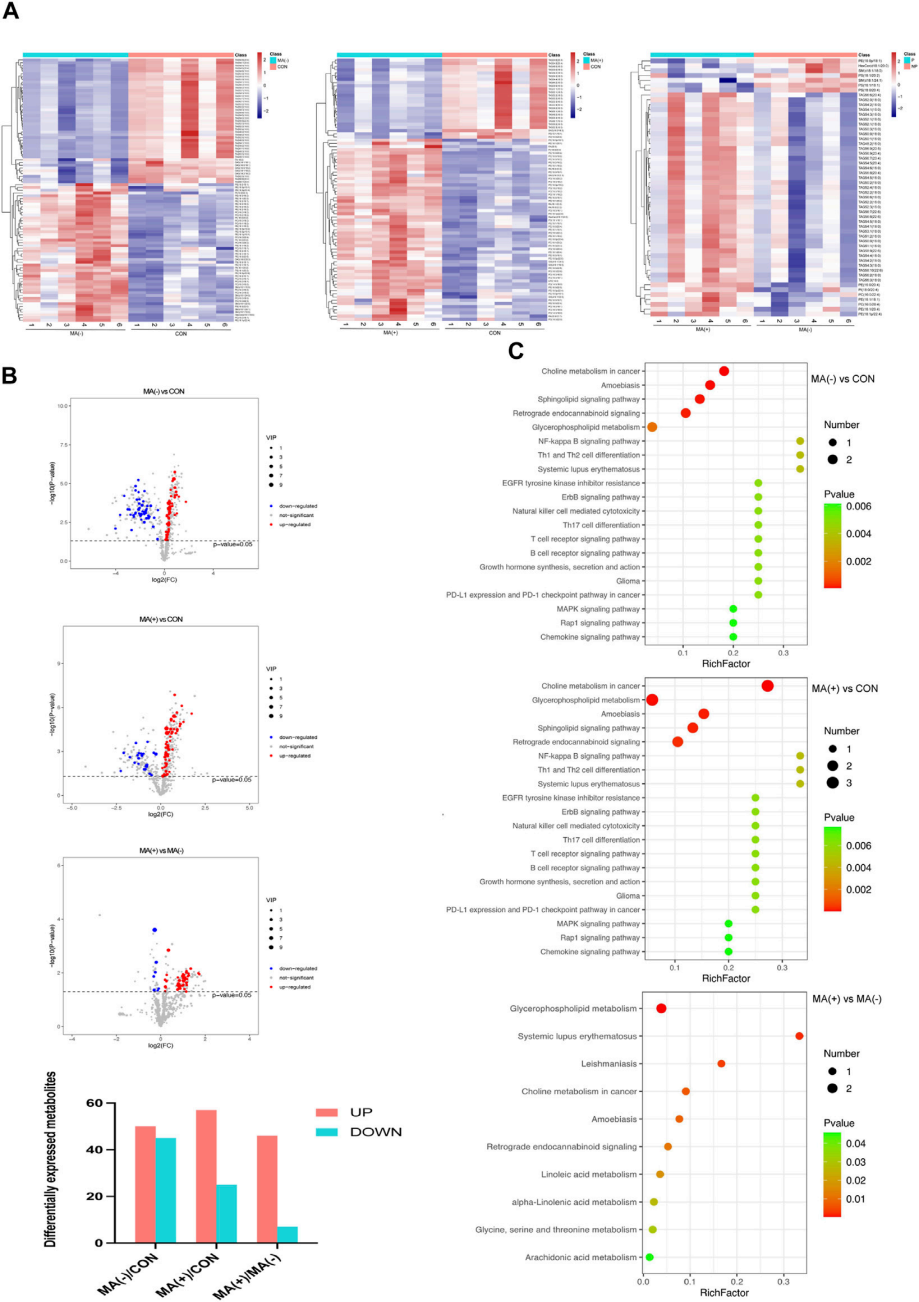
There were more elevated lipid metabolites in the MA (+) group. **(A)**. Heat map of differentially expressed metabolites hierarchical Clustering between different groups (n = 6). The color ranges from blue to red means low to high abundance of metabolite expression. **(B)**. Volcano Plot of differentially expressed metabolites between different groups. Red origin points mean significantly upregulated metabolites, blue origin points mean significantly downregulated metabolites, and gray points mean insignificant metabolites. **(C)**. Pathway enrichment map of differentially expressed metabolites between different groups. Green to red means a sequential decrease in *p*-value. The spot size increases mean the number of metabolites enriched in this pathway increases.

To understand the lipid changes in each group, we examined the content of various lipid metabolites by untargeted lipidomics analysis. The heat map shows that both the MA (−) and MA (+) groups showed disturbed lipid metabolism compared to the control group. Moreover, the MA (+) group showed a higher expression of lipids. Comparing the MA (+) and MA (−) groups individually, similar results could be obtained (Figure 2A). Compared to the control group, 50 significantly expressed upregulated and 45 significantly expressed downregulated lipid metabolites were detected in the MA (−) group, 57 significantly expressed upregulated, and 25 significantly expressed downregulated lipid metabolites were detected in the MA (+) group. Also, 46 significantly expressed upregulated lipid metabolites and 7 significantly expressed downregulated lipid metabolites appeared in MA (+) group relative to MA (−) group (Figure 2B). KEGG enrichment map revealed that differentially expressed metabolites were mainly enriched in choline metabolism in cancer, amoebiasis, sphingolipid signaling pathway, retrograde endocannabinoid signaling, glycerophospholipid metabolism (Figure 2C: MA (−) vs CON). And choline metabolism in cancer, glycerophospholipid metabolism, amoebiasis, sphingolipid signaling pathway, and retrograde endocannabinoid signaling (Figure 2C: MA (+) vs CON). Differentially expressed metabolites between MA (+) and MA (−) groups were mainly enriched in metabolism-related pathways, including glycerophospholipid metabolism, choline metabolism in cancer, and linoleic acid metabolism (Figure 2C: MA (+) vs MA (−)). These results indicate that more severe lipid accumulation occurred in the MA (+) group, which may be associated with glycerophospholipid metabolism, choline metabolism in cancer, and the linoleic acid metabolism pathway.

The development of MA is associated with lipid droplet formation and myelin degeneration.

**FIGURE 3:**
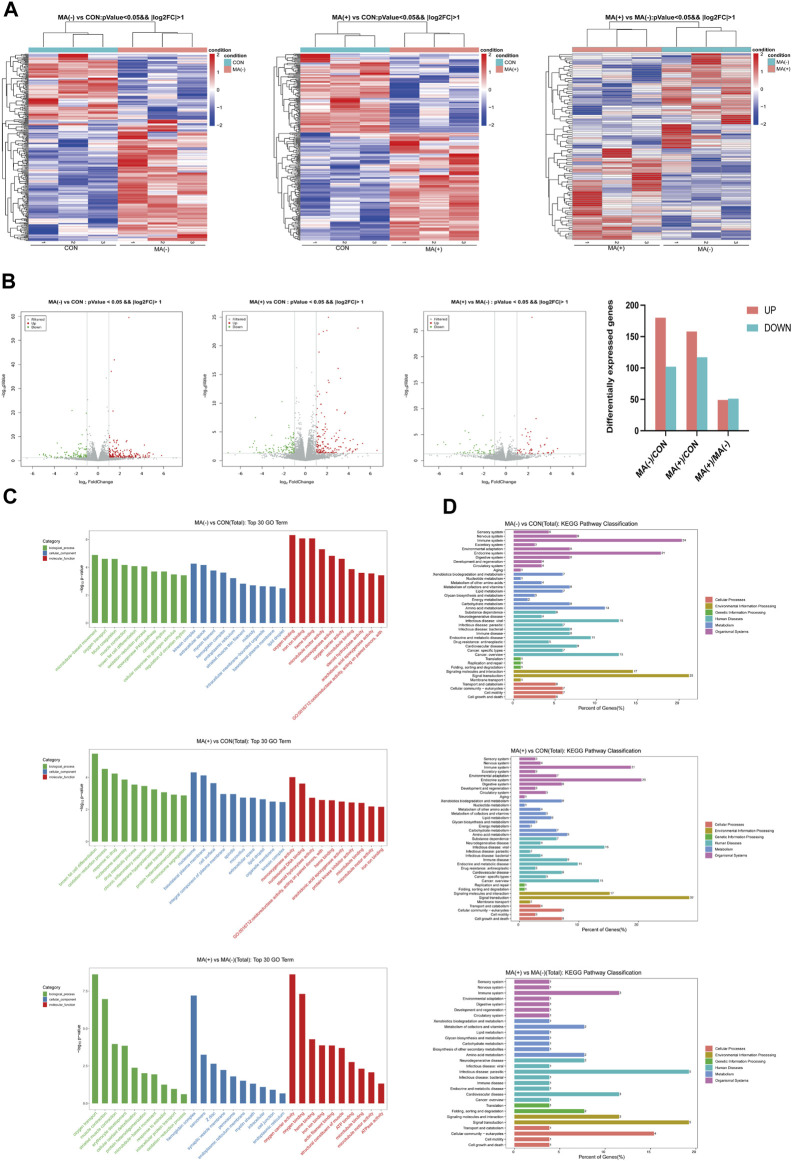
Changes in gene expression levels were observed in the MA (−) and MA (+) groups. **(A)**. Heat map of differentially expressed gene hierarchical Clustering between different groups (n = 3). The color ranges from blue to red means low to high gene expression abundance. **(B)**. Volcano Plot of differentially expressed genes between different groups. Red origin points mean significantly upregulated genes, green origin points mean significantly downregulated genes, and gray points mean insignificant genes. **(C)**. GO enrichment analysis of differentially expressed genes between different groups. **(D)**. KEGG enrichment analysis of differentially expressed genes between different groups.

To understand the alteration of genes in each group at the transcriptional level, we examined the mRNA expression levels of each group and performed transcriptomic analysis. From the heat map, it can be found that the expression level of the mRNA was altered in the MA (−) and MA (+) groups compared to the control group, and there were also apparent differences between the MA (+) group and the MA (−) group as well (Figure 3A). Compared to the control group, 180 significantly expressed upregulated and 102 significantly expressed downregulated genes were detected in the MA (−) group, and 158 significantly expressed upregulated and 117 significantly expressed downregulated genes were detected in the MA (+) group. Moreso, 49 significantly expressed upregulated genes and 51 significantly expressed downregulated genes appeared in MA (+) group relative to MA (−) group (Figure 3B). GO enrichment analysis was performed to further characterize differentially expressed genes’ functions. GO enrichment found that the differentially expressed genes in the MA (+) and MA (−) groups compared to the control group could be associated with forming lipid droplets and chronic inflammatory response. The oxidation-reduction process may be related to the occurrence of MA (Figure 3C: MA (−) vs CON, MA (+) vs CON). GO enrichment analysis of differentially expressed genes between the MA (+) and MA (−) groups suggested that the oxidation-reduction process, endoplasmic reticulum membrane, and myelin sheath function may be involved in the development of MA (Figure 3C: MA (+) vs MA (−)). By enriching these differentially expressed genes *via* the KEGG pathway, we found that these pathways are primarily associated with the nervous system, neurodegenerative pathologies, and diverse metabolisms (Figure 3D). Thus, these results suggest that the development of MA may be related to metabolic disturbances of the nervous system.

Elevated expression of phosphatidylcholine may be associated with the downregulation of CYP1A2 expression.

**FIGURE 4:**
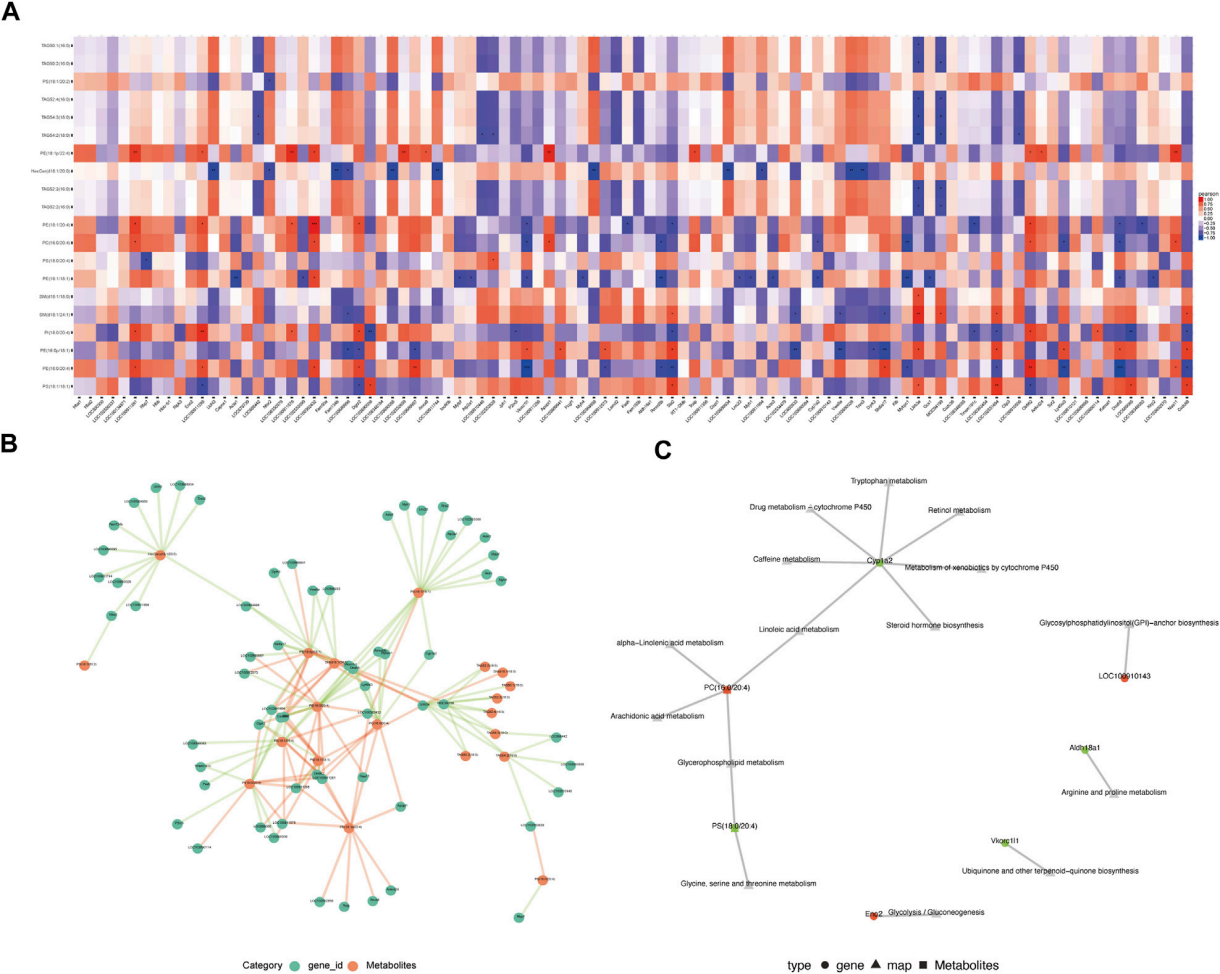
Integrated analysis of lipidomics and transcriptomics. **(A)**. Heat map of the correlation between differentially expressed genes and differentially expressed metabolites. Red is a positive correlation, blue is a negative correlation, and darker color means greater correlation.*p < 0.05, **p < 0.01,***p < 0.001. **(B)**. Association network of differentially expressed genes and differentially expressed metabolites (*p* < 0.05). The red line means a positive correlation, and the green line means a negative correlation. The thickness of the line means the correlation coefficient is high or low. **(C)**. KGML network analysis of differentially expressed genes and differentially expressed metabolites. Red indicates upregulated genes or metabolites, and green indicates downregulated genes or metabolites.

Correlation analysis was performed for differentially expressed metabolites and genes between the MA (−) and MA (+) groups to further explore the mechanisms underlying the development of MA. The R software was used to calculate the Pearson correlation coefficients for differentially expressed metabolites and genes, then draw the correlation heat map of differentially expressed metabolites and genes (Figure 4A). Additionally, Cytoscape software was used to map the correlation network between differentially expressed metabolites and genes (Figure 4B). To investigate transcriptome and metabolome interactions more systematically, we performed a KGML network analysis of differentially expressed metabolites and genes to reflect the network relationship between genes and metabolites (Figure 4C). Therefore, these results indicate that the elevated expression of phosphatidylcholine may be associated with the downregulation of CYP1A2 expression.

The MA (+) group exhibited decreased expression levels of CYP1A2, more severe myelin sheath degeneration, and accumulation of lipid droplets.

**FIGURE 5:**
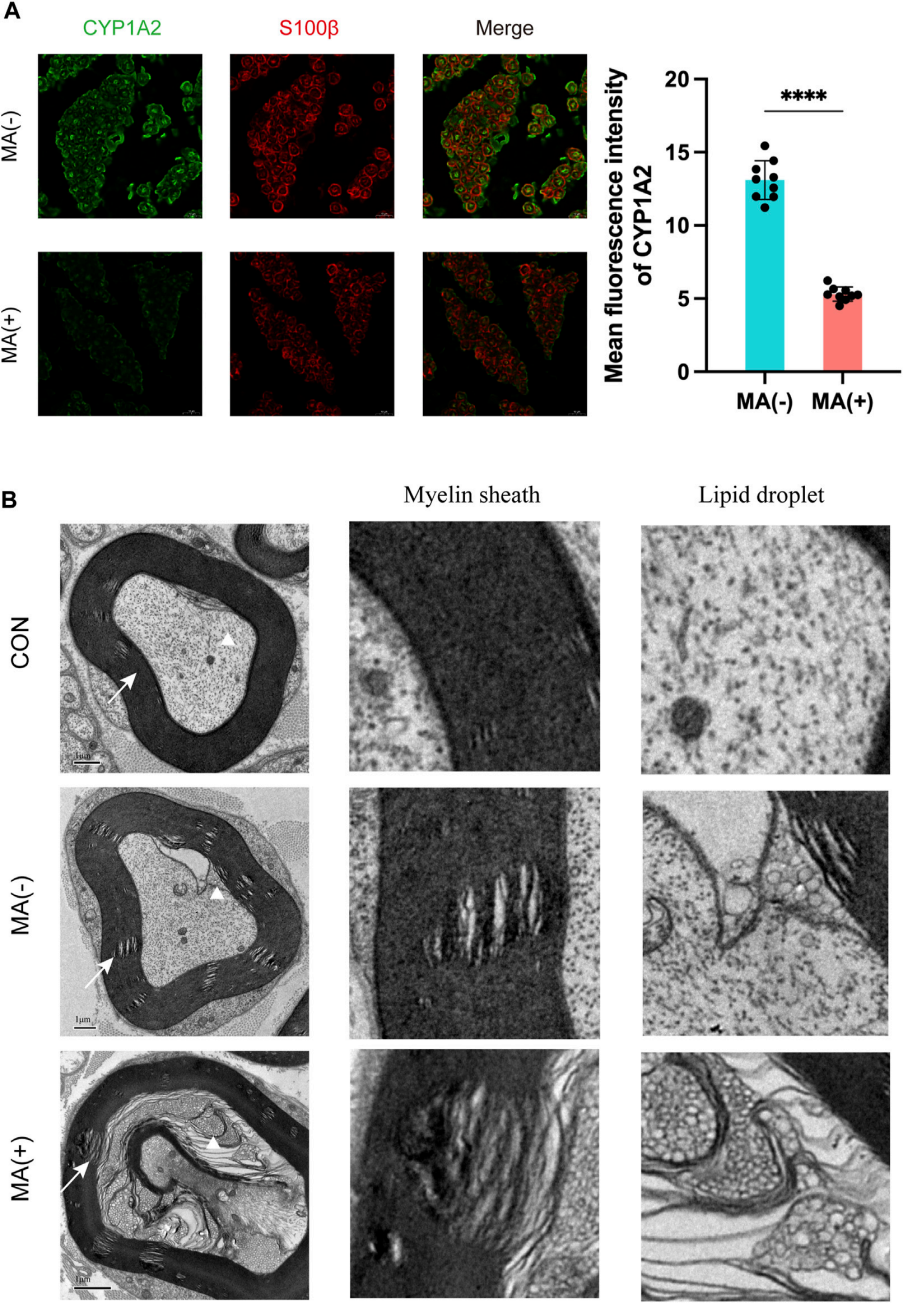
Elevated expression levels of CYP1A2 protein, degeneration of myelin sheath, and lipid droplet accumulation in the sciatic nerve in the MA (+) group. **(A)**. Immunofluorescence staining with anti-CYP1A2 (green) and anti-S100β(red) and quantification showing decreased fluorescence intensity of CYP1A2 in myelinated axons of MA (+) group rats (n = 3). The scale bar is 10 μm. Mean ± SD. Each data point means the value of 1 field examined. **(B)**. Electron microscopy showing degeneration of myelin sheath (indicated by the white arrow) and lipid droplets (indicated by the white triangle) in MA (−) group and MA (+) group rats (n = 3). The scale bar is 1 μm.

**FIGURE 6:**
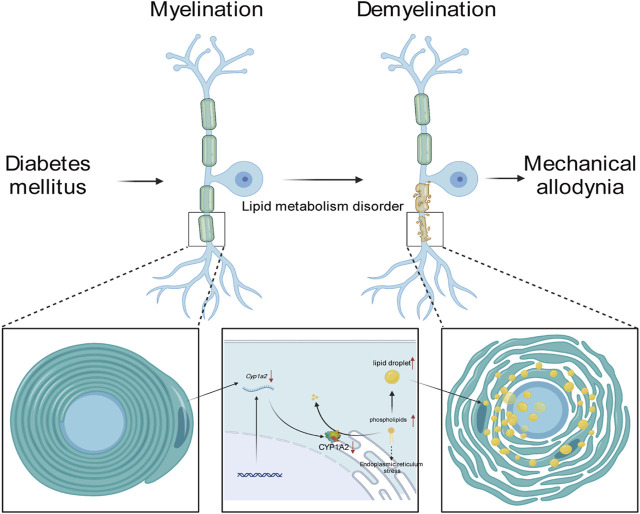
Mechanism of MA regulation by CYP1A2.

Immunofluorescence staining and transmission electron microscopy scanning were performed to further validate the results of the integrated lipidomics and transcriptomics analysis. Immunofluorescence staining showed that the expression level of CYP1A2 was higher in MA (−) group than MA (+) group (Figure 5A). Transmission electron microscopy showed that degeneration of myelin sheath (Figure 5B: white triangle) and accumulation of lipid droplets (Figure 5B: white arrow) were observed in both the MA (−) and MA (+) groups and these were more severe in the MA (+) group. These results support the downregulation of CYP1A2 expression, more severe degeneration of myelin sheath, and accumulation of lipid droplets in the MA (+) group. Therefore, we deduced that the downregulation of CYP1A2 expression led to myelin sheath degeneration and accumulation of lipid droplets in PDPN rats (Figure 6).

## Discussion

In this study, we demonstrated lipid metabolism disorders and transcriptomics alteration in the sciatic nerve of rats in the MA (+) and other groups. To further clarify the regulatory network in the sciatic nerve of rats in the MA (+) group, we performed correlation analysis of differentially expressed metabolites and differentially expressed genes as well as KGML network analysis, and based on the results of the integrated analysis, we predicted that CYP1A2 might be the cause of lipid metabolism disorders in the MA (+) group. IF confirmed the downregulation of CYP1A2 expression in MA (+) group, and more severe lipid droplet accumulation and myelin sheath degeneration that occurred in the MA (+) group was confirmed by transmission electron microscopy. Our study shows that more severe accumulation of lipid droplets and myelin sheath degeneration were found in the sciatic nerve of PDPN rats. Thus, these might be associated with the downregulation of CYP1A2 expression.

Lipidomics analysis showed that the MA (−) and MA (+) groups both had disorders of lipid metabolism, mainly including upregulation of multiple phosphatidylcholines (PC)and phosphatidylethanolamine (PE) lipid expression and downregulation of multiple Triglycerides (TAG) expression. These findings are consistent with the study by Ferdousi and Hua Cheng et al. (Cheng et al., 2006; Ferdousi et al., 2021). Furthermore, more lipid species in the MA (+) group showed upregulation, as shown in Figure 2, suggesting lipid accumulation may cause MA. This finding is similar to previous studies indicating that lipid homeostasis is closely linked to peripheral neuropathy (Cermenati et al., 2015; Freeman et al., 2016; Perez-Matos et al., 2017; O'Brien et al., 2020). Consistent with a previous study (Palavicini et al., 2020), there was no significant difference in the levels of blood glucose between the MA (−) and MA (+) groups, as shown in Figure 1; Supplementary Figure S1, suggesting that MA is primarily associated with hyperlipidemia rather than hyperglycemia. Phosphatidylcholine is synthesized in the ER, which has essential functions, including the provision of membranes required for protein synthesis and export, cholesterol homeostasis, and triacylglycerol storage and secretion (Lagace and Ridgway, 2013). Phosphatidylcholine homeostasis is critical for cells to perform normal physiological functions, and disturbances in phosphatidylcholine metabolism may lead to cellular dysfunction and thus affect sciatic nerve function.

To further elucidate the reason for the upregulation of lipid expression, we performed transcriptome sequencing, which showed that the functions of the differentially expressed genes were primarily linked to the lipid droplet (MA (+) vs CON, MA (−) vs CON) and myelin sheath (MA (+) vs MA (−)) as shown in Figure 3. This suggests that altered expression of genes related to lipid droplets occurred in both diabetic rats and the myelin sheath in the MA (+) group, indicating that lipid overload is closely related to MA. Integrated analysis was performed to understand the network of relationships between altered lipidomics and transcriptomics. Dynamic changes in lipid metabolism within neurons and glial cells resulting in lipid accumulation and lipid droplet formation are present in brain models of various neurodegenerative diseases (Yang et al., 2022), and myelin sheath is essential for the normal function of peripheral nerves. The results suggested that the downregulation of CYP1A2 expression may be responsible for PC accumulation, as shown in Figure 4. Downregulation of CYP1A2 expression in MA (+) was demonstrated by immunofluorescence. Transmission electron microscopy results suggested that the occurrence of lipid droplets was a cause of demyelination and axonal degeneration, as shown in Figure 5. Based on the above findings, we hypothesized that downregulation of CYP1A2 expression results in abnormal PC accumulation in the sciatic nerve of diabetic rats, which ultimately leads to lipid droplets formation, and that the formation of these droplets disrupts the normal morphology of myelin and axons and impairs their function, ultimately resulting in the development of MA.

From the immunofluorescence images (Supplementary Figure S4A), we can see that CYP1A2 is mainly expressed in Schwann cells’ cytosol and medial myelin sheath. The disruption of lipid metabolism in peripheral nerves may have begun first in Schwann cells, consistent with many recent studies on Schwann cells (Nadra et al., 2008; Verheijen et al., 2009; Montani et al., 2018; Meyer Zu Reckendorf et al., 2020). A study showed that an elevated PC/PE ratio impaired ER homeostasis and caused cellular dysfunction (Fu et al., 2011). Another study also demonstrated that elevated 20:4-PC affects the proliferative function of cells (Koeberle et al., 2013). Since similar lipid alterations were seen in our result, these may be another cause of peripheral nerve demyelination in MA rats. Moreover, results of transmission electron microscopy from rats in the MA (+) group showed (Supplementary Figure S4B) that accumulation of lipid droplets and degeneration of myelin sheath occurred primarily in large myelinated fibers (Aβ fibers). According to the gate control theory of pain (Melzack and Wall, 1965; Wall, 1978), Aβ is associated mainly with inhibitory afferents of neuronal signaling. Furthermore, damage in Aβ fibers can block inhibitory nerve action potential afferents, leading to nociceptive hyperalgesia and MA.

In the present study, we investigated the relationship between PDPN and lipid metabolism for the first time. We applied a multi-omics study of lipidomics and transcriptomics in the sciatic nerve of rats, which was verified by immunofluorescence and transmission electron microscopy. We deduce that abnormal CYP1A2 expression disrupts PC lipid metabolism, which might lead to lipid droplet formation and myelin degeneration, ultimately leading to the development of MA in PDPN rats. There are also some limitations to our study, such as how CYP1A2 specifically affects PC metabolism, whether altered expression levels of CYP1A2 have a direct effect on Schwann cell function leading to MA, and whether TAG produced different decreases in the MA (−) and MA (+) groups related to MA, all of which need further investigation. This study used type 1 diabetic rats, and further studies are needed to determine whether our results are consistent with type 2 diabetic rats and human diabetics.

In summary, our results imply that the downregulation of CYP1A2 expression leads to PC lipid disorder and lipid droplet accumulation, and myelin sheath degeneration in PDPN rats. Our study explored the relationship between lipid homeostasis and PDPN for the first time, identified severe lipid droplet accumulation and myelin degeneration in PDPN sciatic nerves, illustrated the importance of dysfunctional lipid metabolism in PDPN, and contributed to our understanding of PDPN.

## Data Availability

The data presented in this study are deposited in the National Center for Biotechnology Information (NCBI) database with the Gene Expression Omnibus (GEO) accession number GSE226315.

## References

[B1] Adibhatla R. M. Hatcher J. F. (2008). Altered lipid metabolism in brain injury and disorders. Subcell. Biochem. 49, 241–268. 10.1007/978-1-4020-8831-5_9 18751914 PMC2293298

[B2] Cermenati G. Audano M. Giatti S. Carozzi V. Porretta-Serapiglia C. Pettinato E. (2015). Lack of sterol regulatory element binding factor-1c imposes glial Fatty Acid utilization leading to peripheral neuropathy. Cell Metab. 21 (4), 571–583. 10.1016/j.cmet.2015.02.016 25817536

[B3] Chaplan S. R. Bach F. W. Pogrel J. W. Chung J. M. Yaksh T. L. (1994). Quantitative assessment of tactile allodynia in the rat paw. J. Neurosci. Methods 53 (1), 55–63. 10.1016/0165-0270(94)90144-9 7990513

[B4] Cheng H. Guan S. Han X. (2006). Abundance of triacylglycerols in ganglia and their depletion in diabetic mice: Implications for the role of altered triacylglycerols in diabetic neuropathy. J. Neurochem. 97 (5), 1288–1300. 10.1111/j.1471-4159.2006.03794.x 16539649 PMC2137160

[B5] Deuis J. R. Dvorakova L. S. Vetter I. (2017). Methods used to evaluate pain behaviors in rodents. Front. Mol. Neurosci. 10, 284. 10.3389/fnmol.2017.00284 28932184 PMC5592204

[B6] Elafros M. A. Andersen H. Bennett D. L. Savelieff M. G. Viswanathan V. Callaghan B. C. (2022). Towards prevention of diabetic peripheral neuropathy: Clinical presentation, pathogenesis, and new treatments. Lancet Neurol. 21 (10), 922–936. 10.1016/S1474-4422(22)00188-0 36115364 PMC10112836

[B7] Ferdousi M. Kalteniece A. Azmi S. Petropoulos I. N. Ponirakis G. Alam U. (2021). Diagnosis of neuropathy and risk factors for corneal nerve loss in type 1 and type 2 diabetes: A corneal confocal microscopy study. Diabetes Care 44 (1), 150–156. 10.2337/dc20-1482 33144353 PMC7783929

[B8] Freeman O. J. Unwin R. D. Dowsey A. W. Begley P. Ali S. Hollywood K. A. (2016). Metabolic dysfunction is restricted to the sciatic nerve in experimental diabetic neuropathy. Diabetes 65 (1), 228–238. 10.2337/db15-0835 26470786

[B9] Fu S. Yang L. Li P. Hofmann O. Dicker L. Hide W. (2011). Aberrant lipid metabolism disrupts calcium homeostasis causing liver endoplasmic reticulum stress in obesity. Nature 473 (7348), 528–531. 10.1038/nature09968 21532591 PMC3102791

[B10] Hagedorn J. M. Engle A. M. George T. K. Karri J. Abdullah N. Ovrom E. (2022). An overview of painful diabetic peripheral neuropathy: Diagnosis and treatment advancements. Diabetes Res. Clin. Pract. 188, 109928. 10.1016/j.diabres.2022.109928 35580704

[B11] Ingelman-Sundberg M. (2004). Pharmacogenetics of cytochrome P450 and its applications in drug therapy: The past, present and future. Trends Pharmacol. Sci. 25 (4), 193–200. 10.1016/j.tips.2004.02.007 15063083

[B12] Koeberle A. Shindou H. Koeberle S. C. Laufer S. A. Shimizu T. Werz O. (2013). Arachidonoyl-phosphatidylcholine oscillates during the cell cycle and counteracts proliferation by suppressing Akt membrane binding. Proc. Natl. Acad. Sci. U. S. A. 110 (7), 2546–2551. 10.1073/pnas.1216182110 23359699 PMC3574958

[B13] Kwon Y. J. Shin S. Chun Y. J. (2021). Biological roles of cytochrome P450 1A1, 1A2, and 1B1 enzymes. Arch. Pharm. Res. 44 (1), 63–83. 10.1007/s12272-021-01306-w 33484438

[B14] Lagace T. A. Ridgway N. D. (2013). The role of phospholipids in the biological activity and structure of the endoplasmic reticulum. Biochim. Biophys. Acta 1833 (11), 2499–2510. 10.1016/j.bbamcr.2013.05.018 23711956

[B15] Liao C. Yang M. Liu P. Zhong W. Zhang W. (2018). Stable rat model of mechanical allodynia in diabetic peripheral neuropathy: The role of nerve compression. J. Reconstr. Microsurg 34 (4), 264–269. 10.1055/s-0037-1621723 29396983

[B16] Lu B. Bridges D. Yang Y. Fisher K. Saltiel A. R. Chang L. (2014). Metabolic crosstalk: Molecular links between glycogen and lipid metabolism in obesity. Diabetes 63 (9), 2935–2948. 10.2337/db13-1531 24722244 PMC4141363

[B17] Melzack R. Wall P. D. (1965). Pain mechanisms: A new theory. Science 150 (3699), 971–979. 10.1126/science.150.3699.971 5320816

[B18] Meyer Zu Reckendorf S. Brand C. Pedro M. T. Hegler J. Schilling C. S. Lerner R. (2020). Lipid metabolism adaptations are reduced in human compared to murine Schwann cells following injury. Nat. Commun. 11 (1), 2123. 10.1038/s41467-020-15915-4 32358558 PMC7195462

[B19] Montani L. Pereira J. A. Norrmén C. Pohl H. B. F. Tinelli E. Trtzmüller M. (2018). De novo fatty acid synthesis by Schwann cells is essential for peripheral nervous system myelination. J. Cell Biol. 217, 1353–1368. jcb.201706010. 10.1083/jcb.201706010 29434029 PMC5881495

[B20] Morgenstern J. Groener J. B. Jende J. M. E. Kurz F. T. Strom A. Gopfert J. (2021). Neuron-specific biomarkers predict hypo- and hyperalgesia in individuals with diabetic peripheral neuropathy. Diabetologia 64 (12), 2843–2855. 10.1007/s00125-021-05557-6 34480211 PMC8563617

[B21] Nadra K. de Preux Charles A. S. Medard J. J. Hendriks W. T. Han G. S. Gres S. (2008). Phosphatidic acid mediates demyelination in Lpin1 mutant mice. Genes Dev. 22 (12), 1647–1661. 10.1101/gad.1638008 18559480 PMC2428062

[B22] O'Brien P. D. Guo K. Eid S. A. Rumora A. E. Hinder L. M. Hayes J. M. (2020). Integrated lipidomic and transcriptomic analyses identify altered nerve triglycerides in mouse models of prediabetes and type 2 diabetes. Dis. Model Mech. 13 (2), dmm042101. 10.1242/dmm.042101 31822493 PMC6994925

[B23] Palavicini J. P. Chen J. Wang C. Wang J. Qin C. Baeuerle E. (2020). Early disruption of nerve mitochondrial and myelin lipid homeostasis in obesity-induced diabetes. JCI Insight 5 (21), e137286. 10.1172/jci.insight.137286 33148881 PMC7710310

[B24] Perez-Matos M. C. Morales-Alvarez M. C. Mendivil C. O. (2017). Lipids: A suitable therapeutic target in diabetic neuropathy? J. Diabetes Res. 2017, 6943851. 10.1155/2017/6943851 28191471 PMC5278202

[B25] Said G. (2007). Diabetic neuropathy--a review. Nat. Clin. Pract. Neurol. 3 (6), 331–340. 10.1038/ncpneuro0504 17549059

[B26] Sun H. Saeedi P. Karuranga S. Pinkepank M. Ogurtsova K. Duncan B. B. (2022). IDF Diabetes Atlas: Global, regional and country-level diabetes prevalence estimates for 2021 and projections for 2045. Diabetes Res. Clin. Pract. 183, 109119. 10.1016/j.diabres.2021.109119 34879977 PMC11057359

[B27] Verheijen M. H. G. Camargo N. Verdier V. Nadra K. de Preux Charles A.-S. Médard J.-J. (2009). SCAP is required for timely and proper myelin membrane synthesis. Proc. Natl. Acad. Sci. 106(50), 21383–21388. 10.1073/pnas.0905633106 19948958 PMC2795508

[B28] Wall P. D. (1978). The gate control theory of pain mechanisms. A re-examination and re-statement. Brain 101 (1), 1–18. 10.1093/brain/101.1.1 205314

[B29] Wang Z. Gerstein M. Snyder M. (2009). RNA-seq: A revolutionary tool for transcriptomics. Nat. Rev. Genet. 10 (1), 57–63. 10.1038/nrg2484 19015660 PMC2949280

[B30] Wishart D. S. (2019). Metabolomics for investigating physiological and pathophysiological processes. Physiol. Rev. 99 (4), 1819–1875. 10.1152/physrev.00035.2018 31434538

[B31] Xicota L. Ichou F. Lejeune F. X. Colsch B. Tenenhaus A. Leroy I. (2019). Multi-omics signature of brain amyloid deposition in asymptomatic individuals at-risk for Alzheimer's disease: The INSIGHT-preAD study. EBioMedicine 47, 518–528. 10.1016/j.ebiom.2019.08.051 31492558 PMC6796577

[B32] Yang D. Wang X. Zhang L. Fang Y. Zheng Q. Liu X. (2022). Lipid metabolism and storage in neuroglia: Role in brain development and neurodegenerative diseases. Cell Biosci. 12 (1), 106. 10.1186/s13578-022-00828-0 35831869 PMC9277953

[B33] Yoon J. H. Seo Y. Jo Y. S. Lee S. Cho E. Cazenave-Gassiot A. (2022). Brain lipidomics: From functional landscape to clinical significance. Sci. Adv. 8 (37), eadc9317. 10.1126/sciadv.adc9317 36112688 PMC9481132

[B34] Zhao Z. J. Zheng R. Z. Wang X. J. Li T. Q. Dong X. H. Zhao C. Y. (2022). Integrating lipidomics and transcriptomics reveals the crosstalk between oxidative stress and neuroinflammation in central nervous system demyelination. Front. Aging Neurosci. 14, 870957. 10.3389/fnagi.2022.870957 35547618 PMC9083465

[B35] Zhou H. Yang X. Liao C. Chen H. Wu Y. Xie B. (2022). The development of mechanical allodynia in diabetic rats revealed by single-cell RNA-seq. Front. Mol. Neurosci. 15, 856299. 10.3389/fnmol.2022.856299 35668789 PMC9165721

