# Supplementary figures and images for: Multi-omics analysis revealed the role of CYP1A2 in the induction of mechanical allodynia in type 1 diabetes
Source: Front Genet. 2023 Mar 23;14:1151340. doi: 10.3389/fgene.2023.1151340 (PMC10076588; doi:10.3389/fgene.2023.1151340)

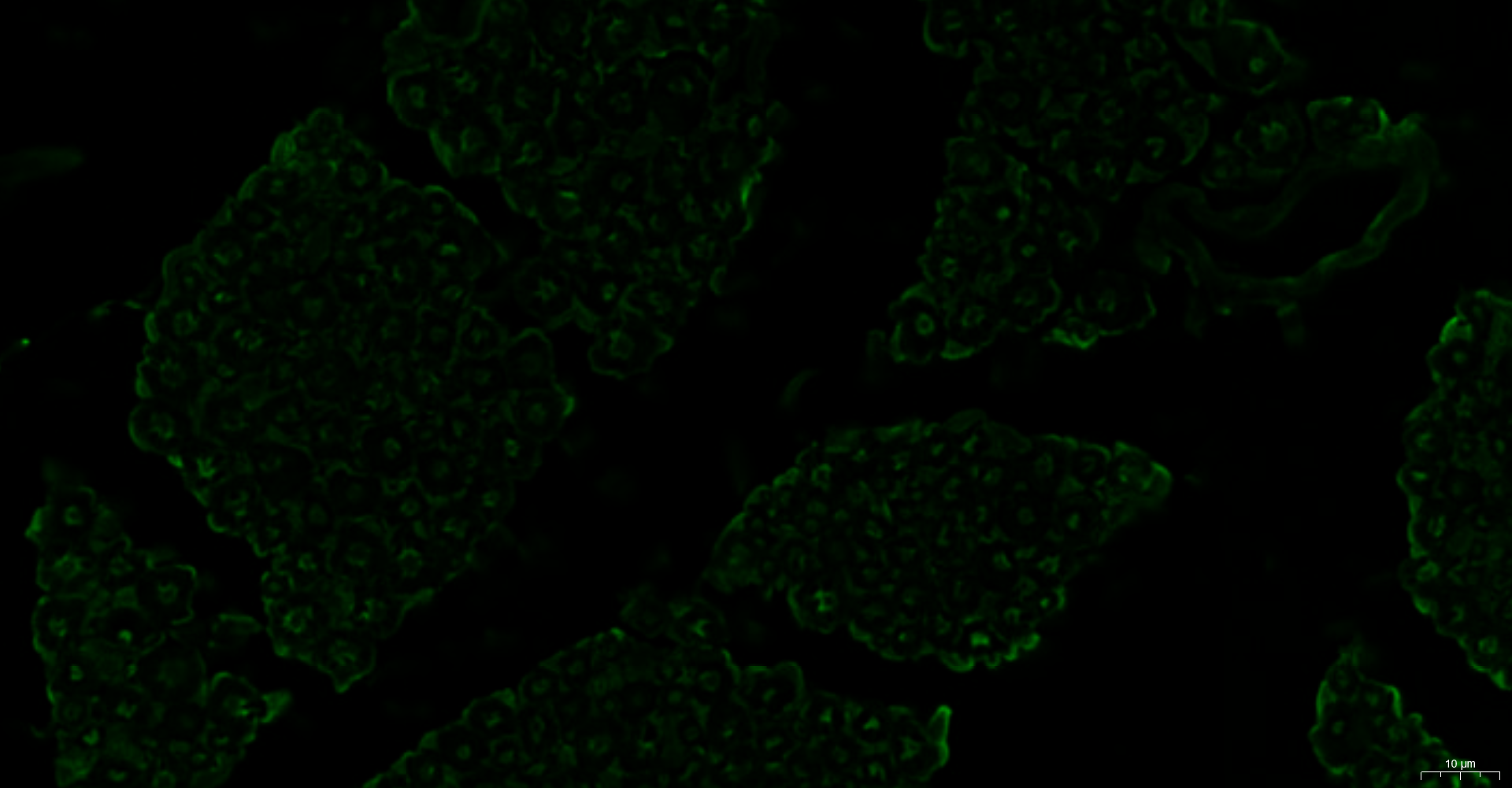

Supplement: Supplementary file 1 [file DataSheet3.ZIP › Immunofluorescence images∩╝êFor review purpose only∩╝ë/MA+/8MA(+)CYP1A2(488)100.0x.tif]

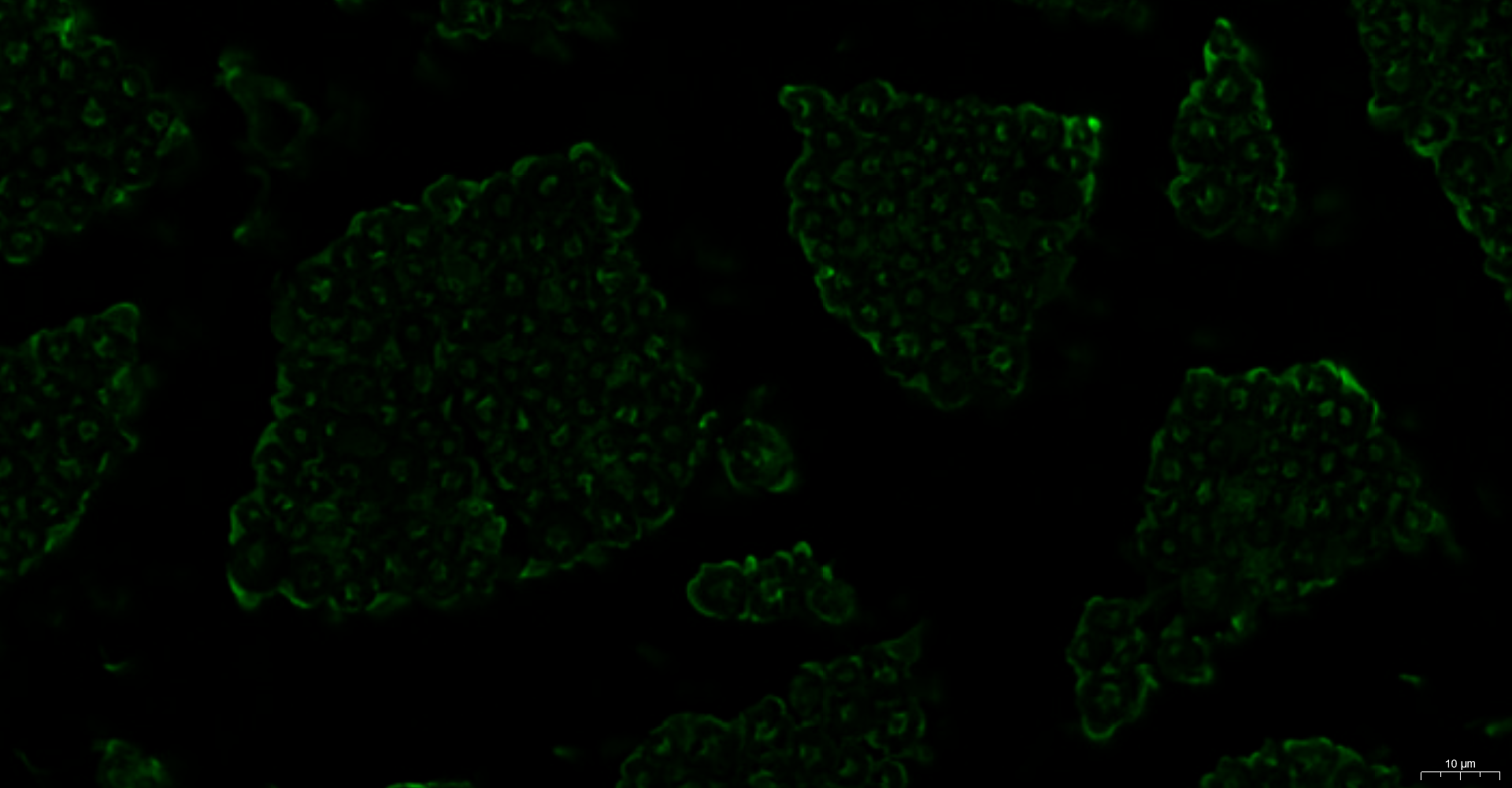

Supplement: Supplementary file 1 [file DataSheet3.ZIP › Immunofluorescence images∩╝êFor review purpose only∩╝ë/MA+/7MA(+)CYP1A2(488)100.0x.tif]

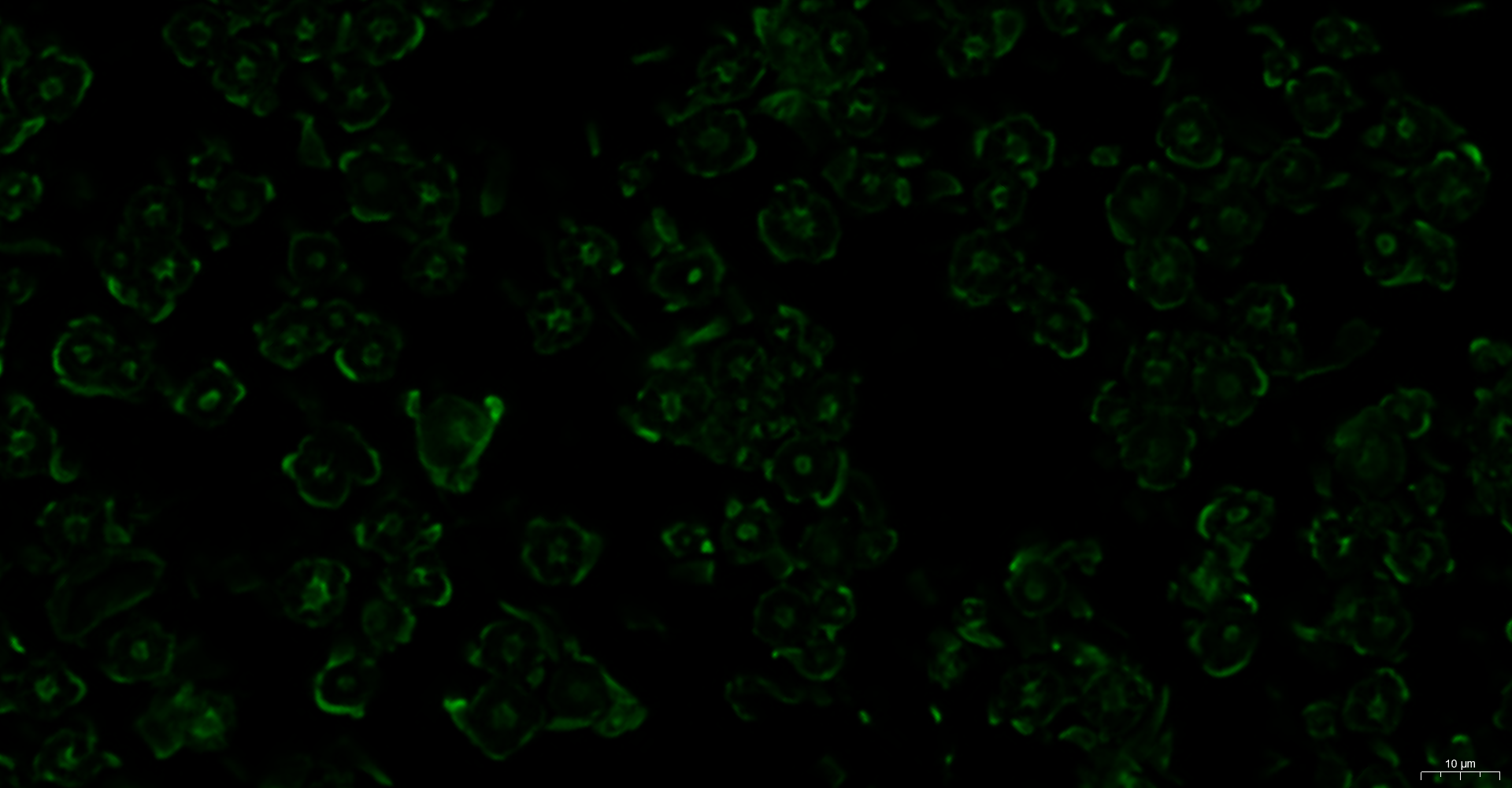

Supplement: Supplementary file 1 [file DataSheet3.ZIP › Immunofluorescence images∩╝êFor review purpose only∩╝ë/MA+/2MA(+)CYP1A2(488)100.0x.tif]

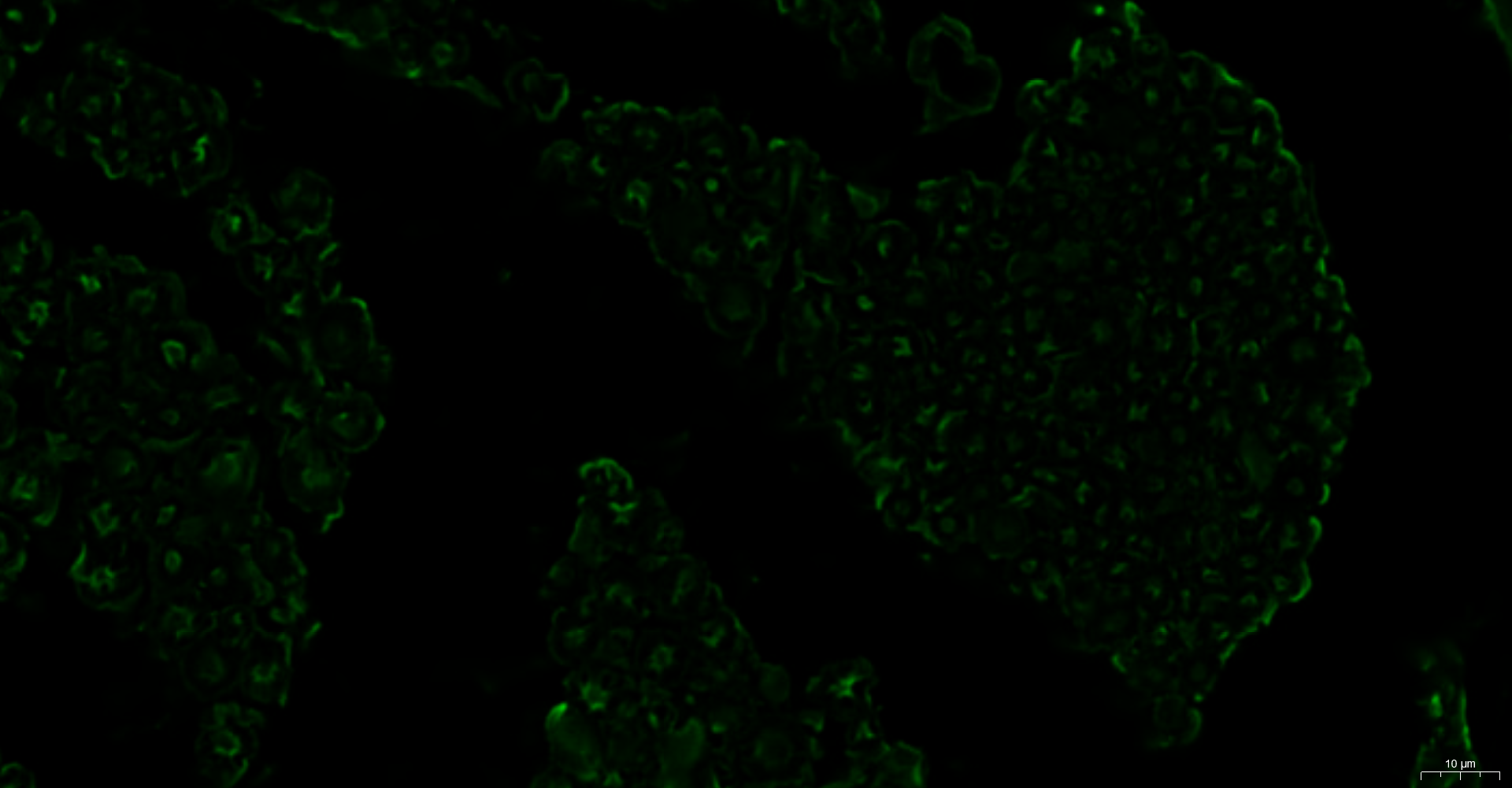

Supplement: Supplementary file 1 [file DataSheet3.ZIP › Immunofluorescence images∩╝êFor review purpose only∩╝ë/MA+/5MA(+)CYP1A2(488)100.0x.tif]

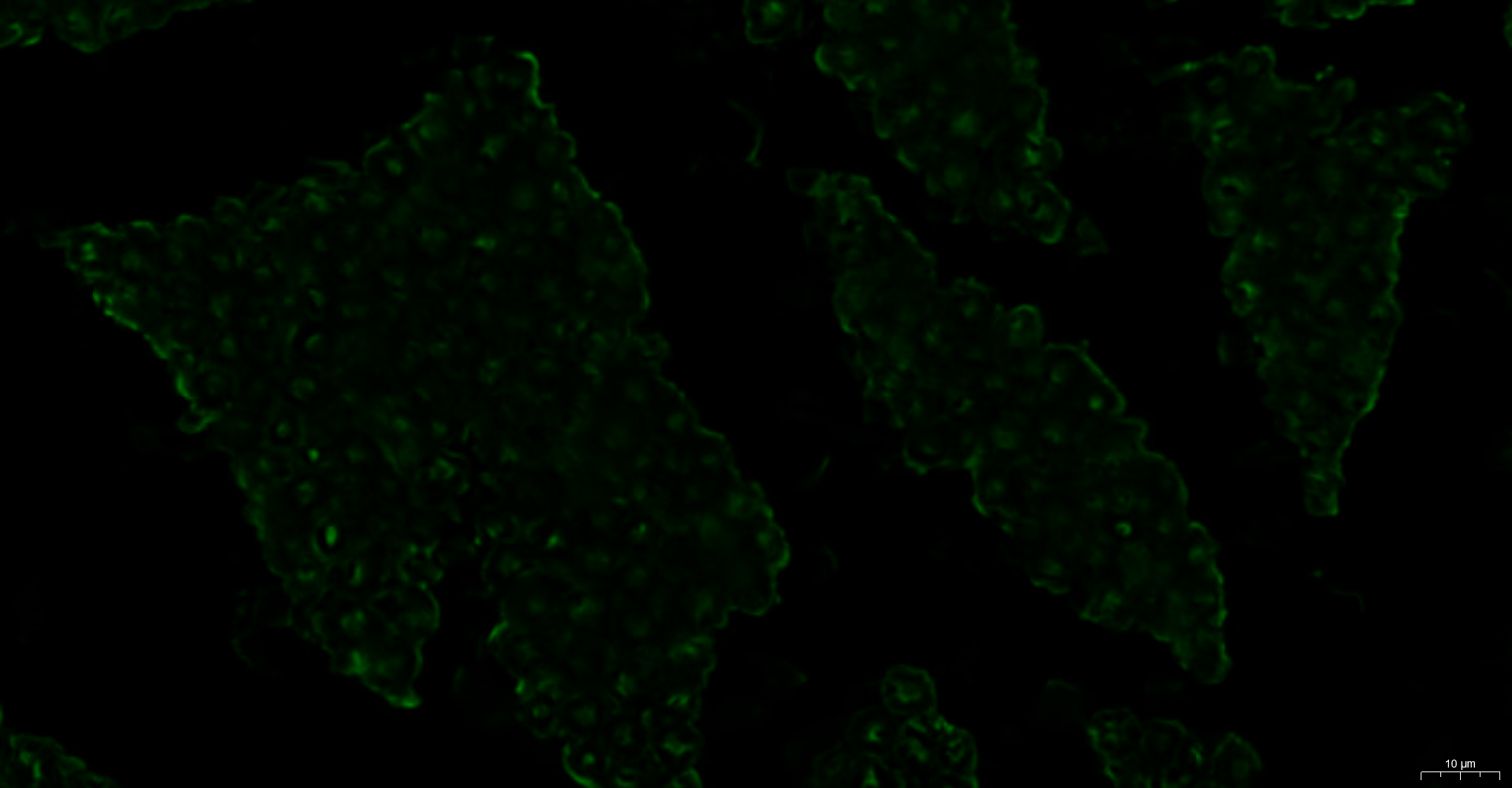

Supplement: Supplementary file 1 [file DataSheet3.ZIP › Immunofluorescence images∩╝êFor review purpose only∩╝ë/MA+/4MA(+)CYP1A2(488)100.0x.tif]

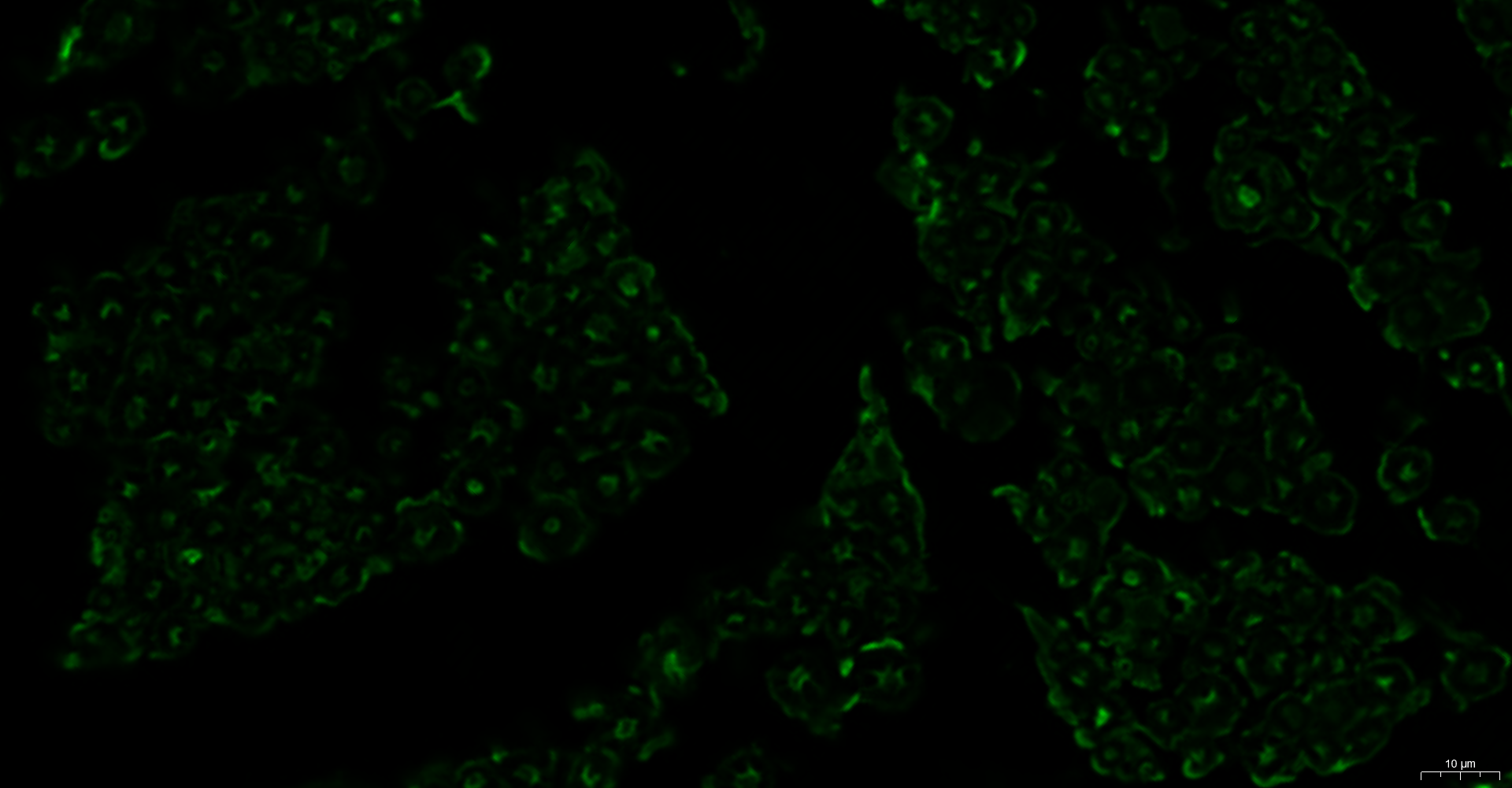

Supplement: Supplementary file 1 [file DataSheet3.ZIP › Immunofluorescence images∩╝êFor review purpose only∩╝ë/MA+/3MA(+)CYP1A2(488)100.0x.tif]

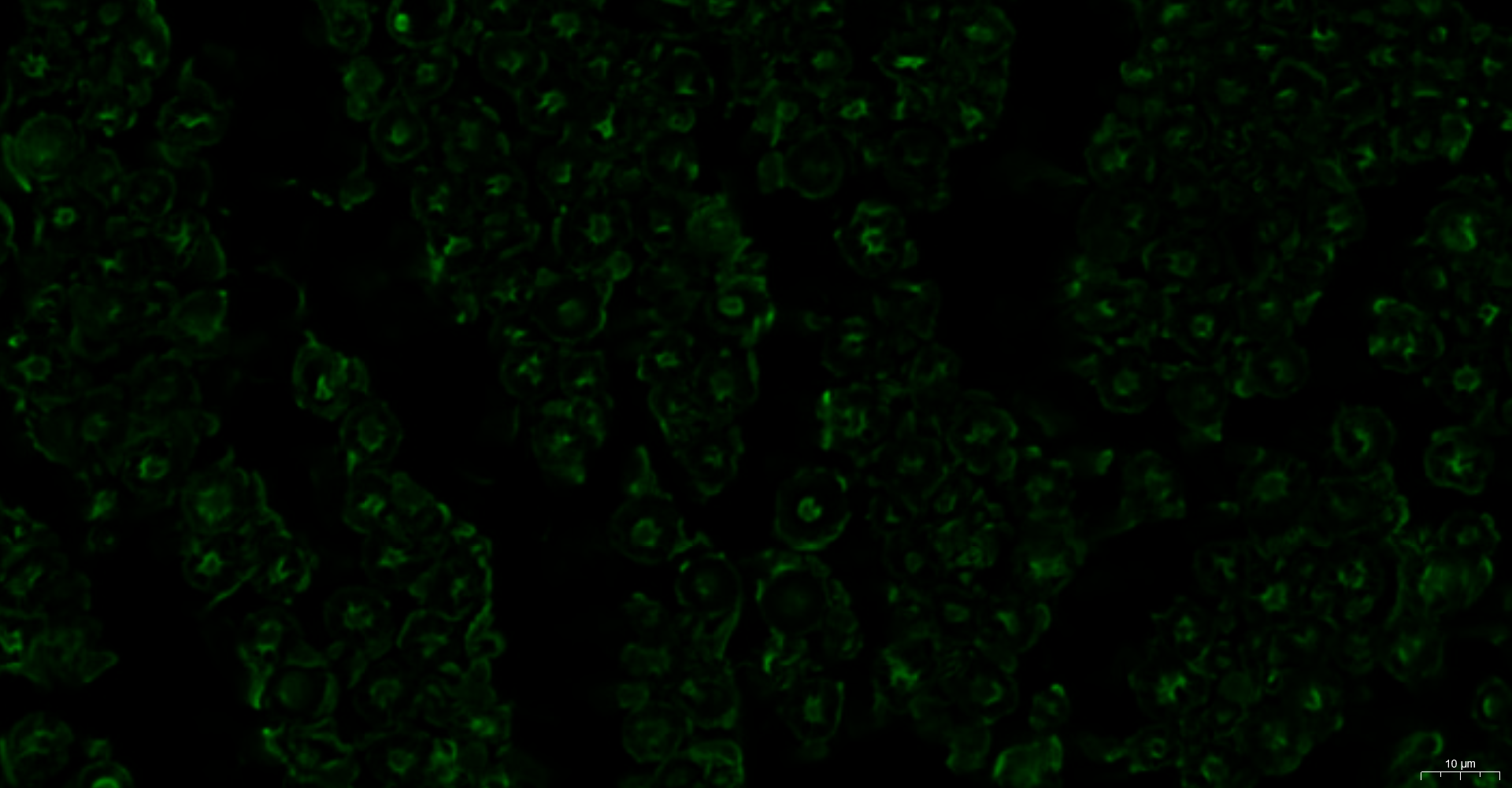

Supplement: Supplementary file 1 [file DataSheet3.ZIP › Immunofluorescence images∩╝êFor review purpose only∩╝ë/MA+/6MA(+)CYP1A2(488)100.0x.tif]

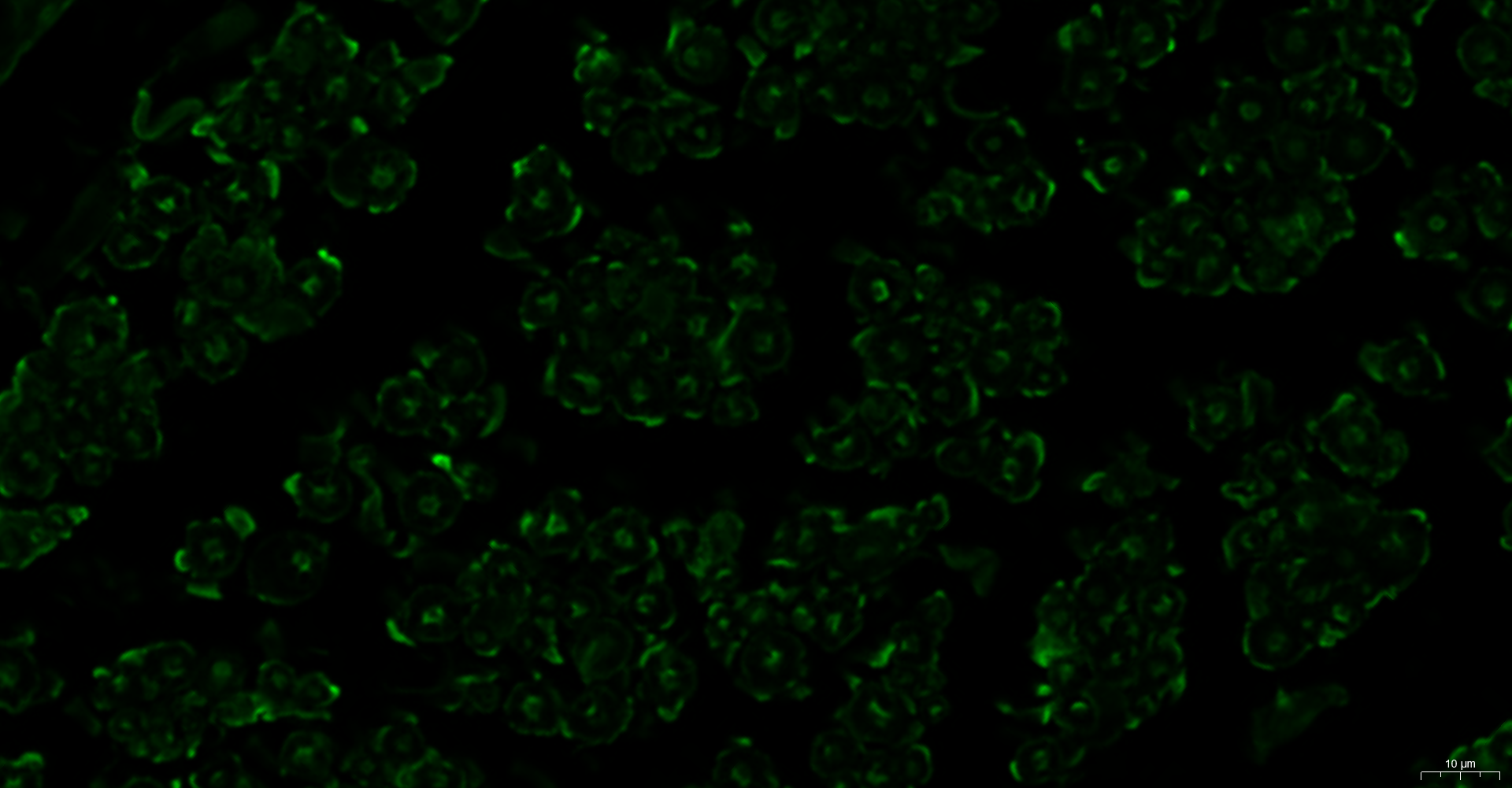

Supplement: Supplementary file 1 [file DataSheet3.ZIP › Immunofluorescence images∩╝êFor review purpose only∩╝ë/MA+/1MA(+)CYP1A2(488)100.0x.tif]

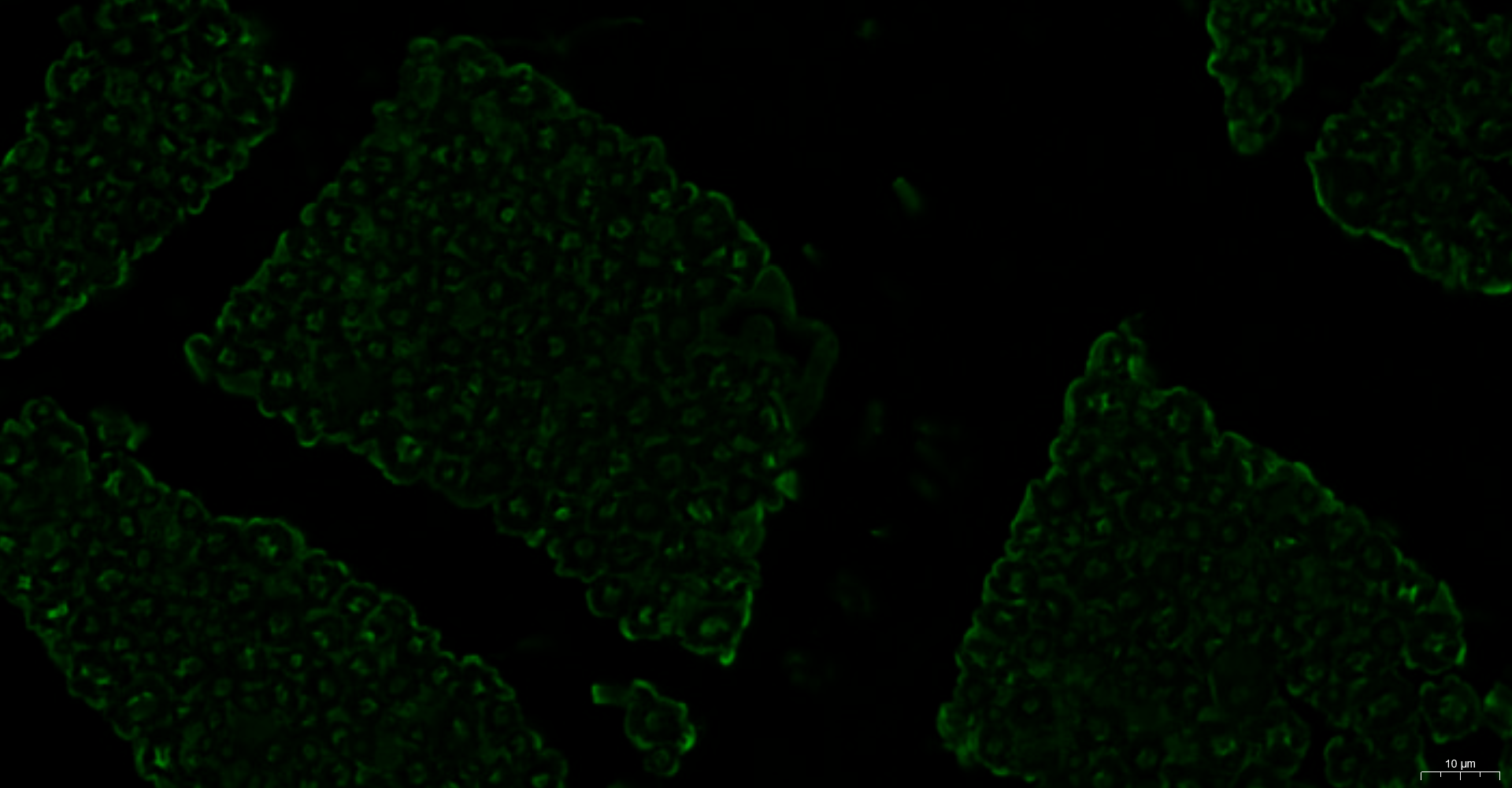

Supplement: Supplementary file 1 [file DataSheet3.ZIP › Immunofluorescence images∩╝êFor review purpose only∩╝ë/MA+/9MA(+) CYP1A2(488)100.0x.tif]

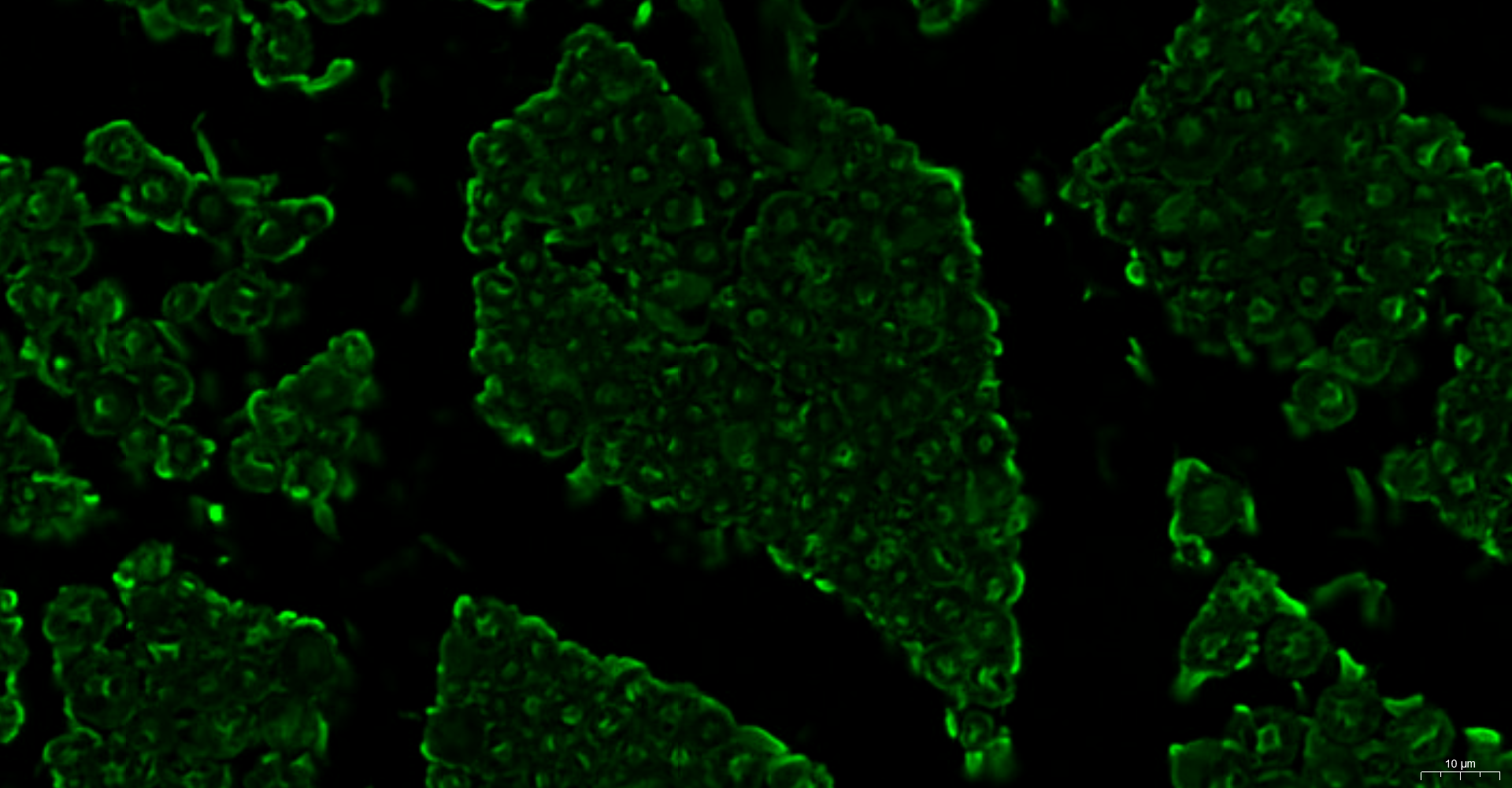

Supplement: Supplementary file 1 [file DataSheet3.ZIP › Immunofluorescence images∩╝êFor review purpose only∩╝ë/MA-/5MA(-)CYP1A2(488)100.0x.tif]

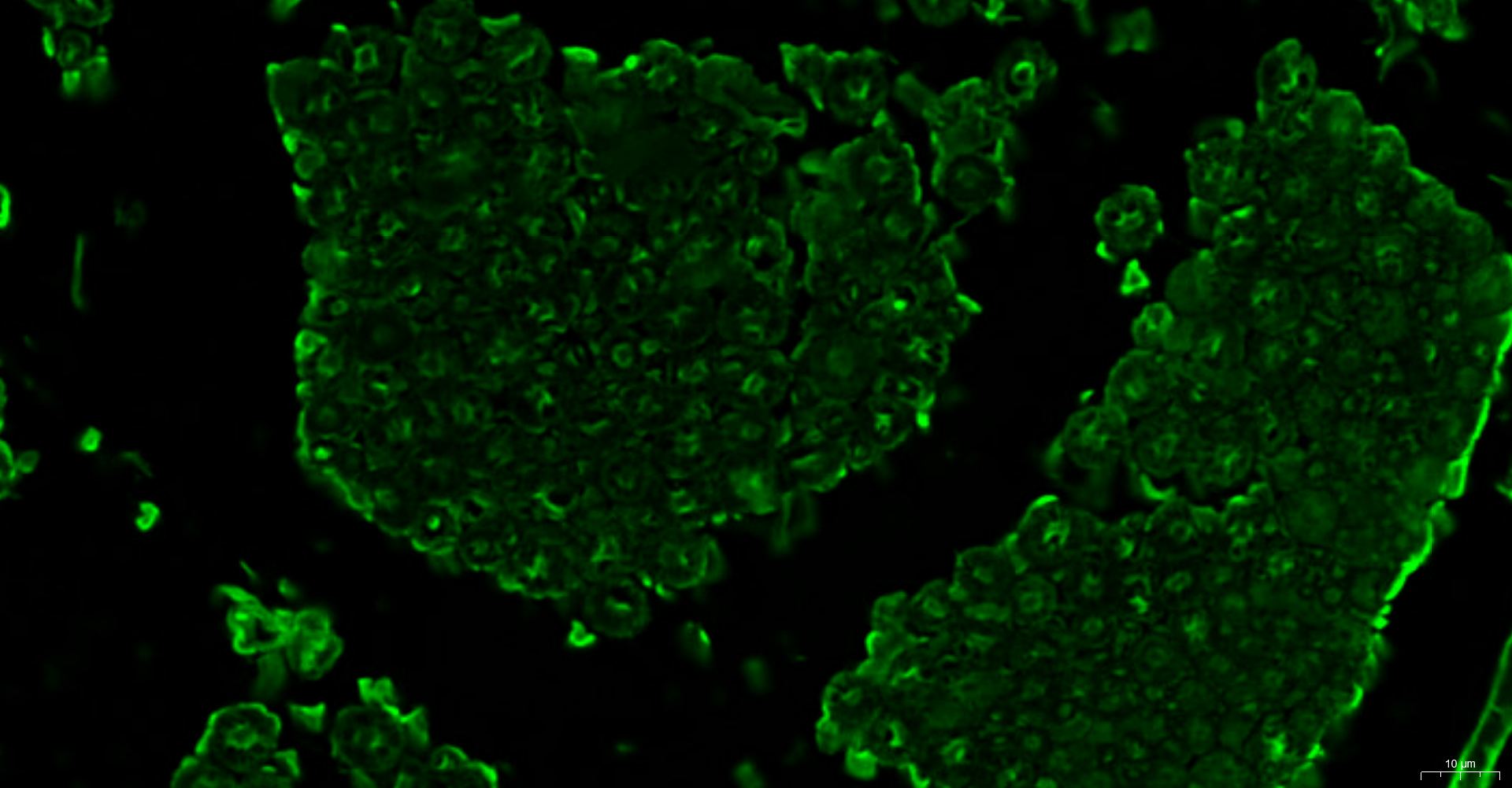

Supplement: Supplementary file 1 [file DataSheet3.ZIP › Immunofluorescence images∩╝êFor review purpose only∩╝ë/MA-/6MA(-) CYP1A2(488)100.0x.tif]

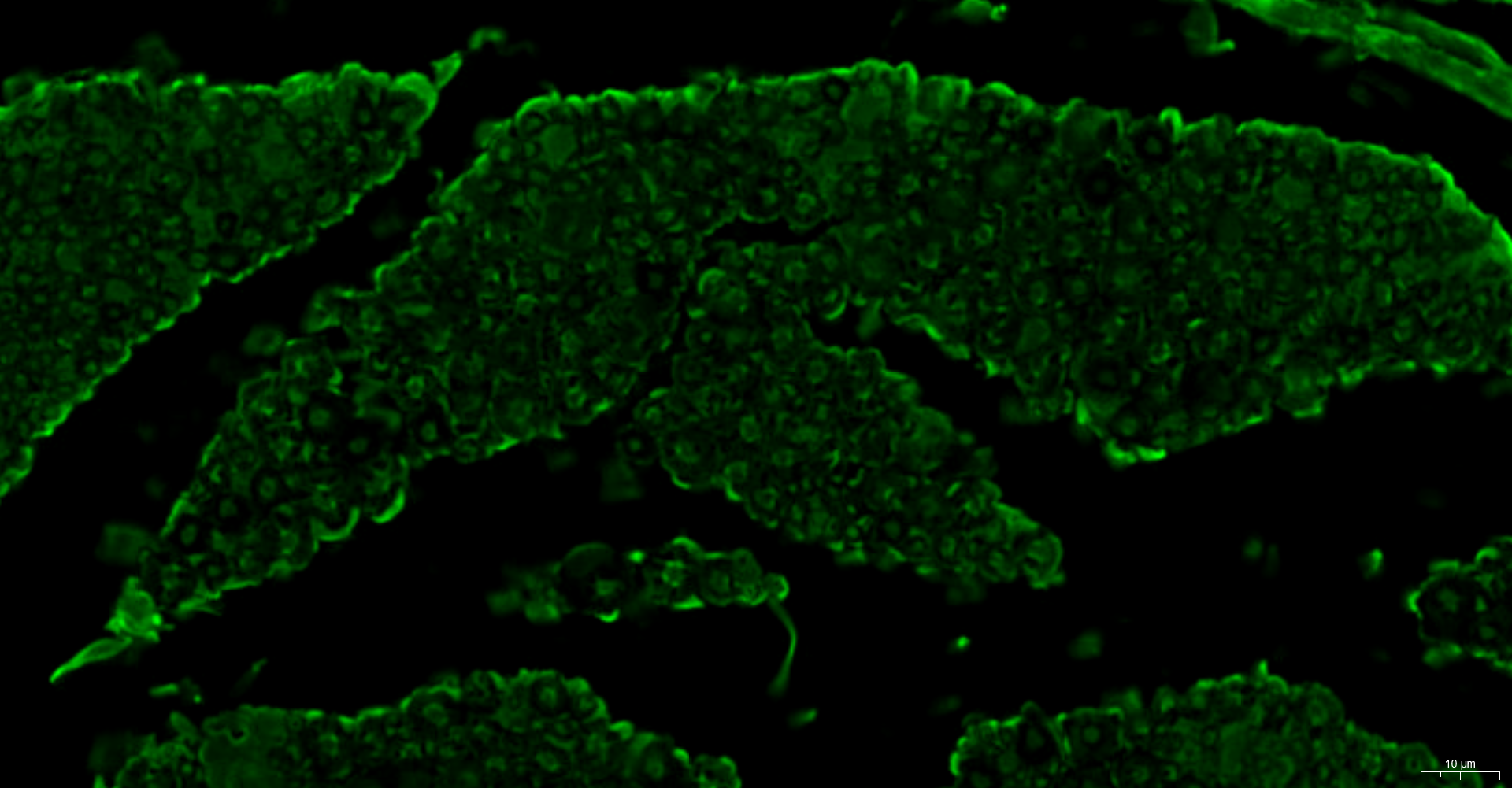

Supplement: Supplementary file 1 [file DataSheet3.ZIP › Immunofluorescence images∩╝êFor review purpose only∩╝ë/MA-/7MA(-)CYP1A2(488)100.0x.tif]

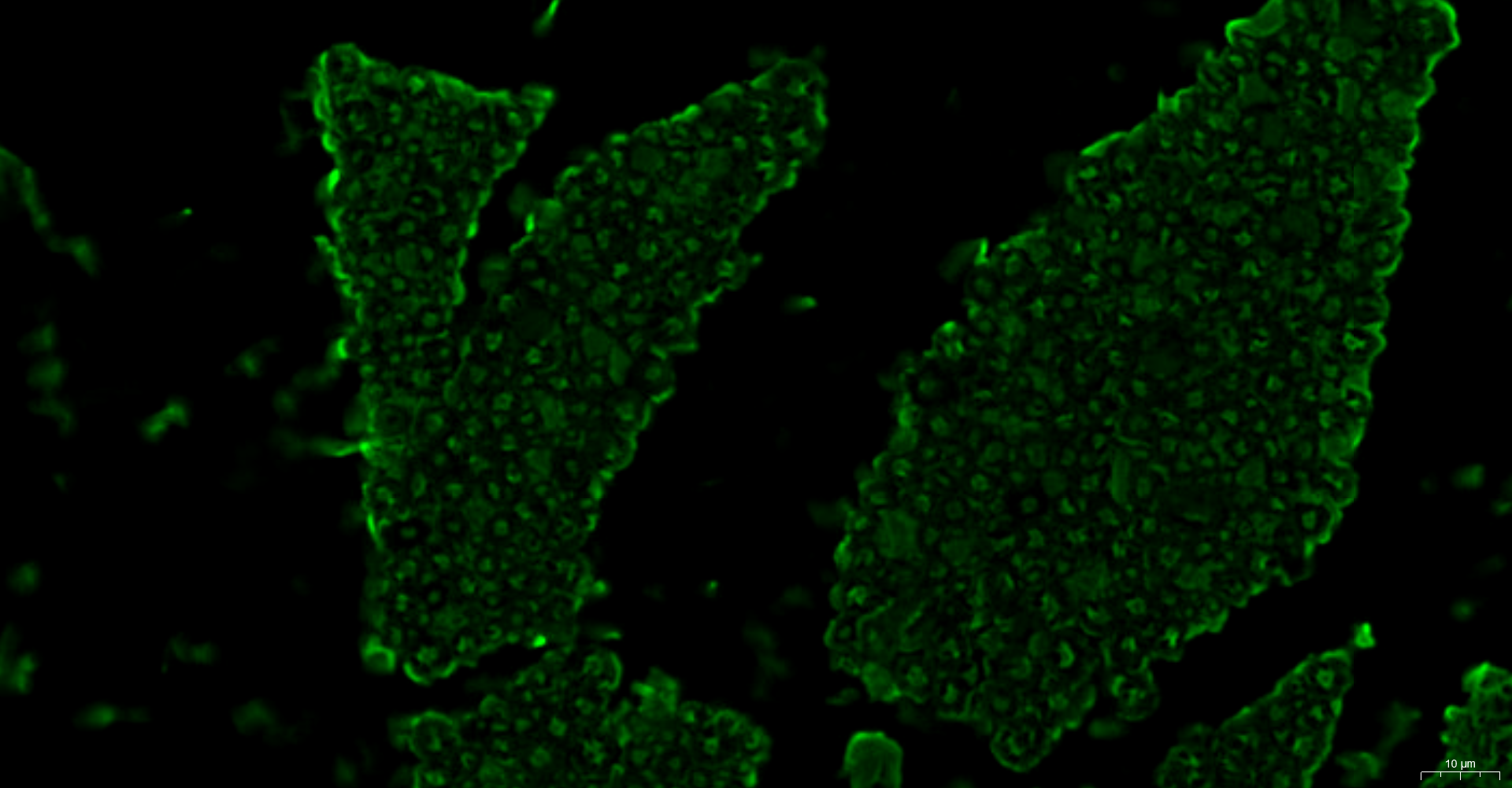

Supplement: Supplementary file 1 [file DataSheet3.ZIP › Immunofluorescence images∩╝êFor review purpose only∩╝ë/MA-/8MA(-)CYP1A2(488)100.0x.tif]

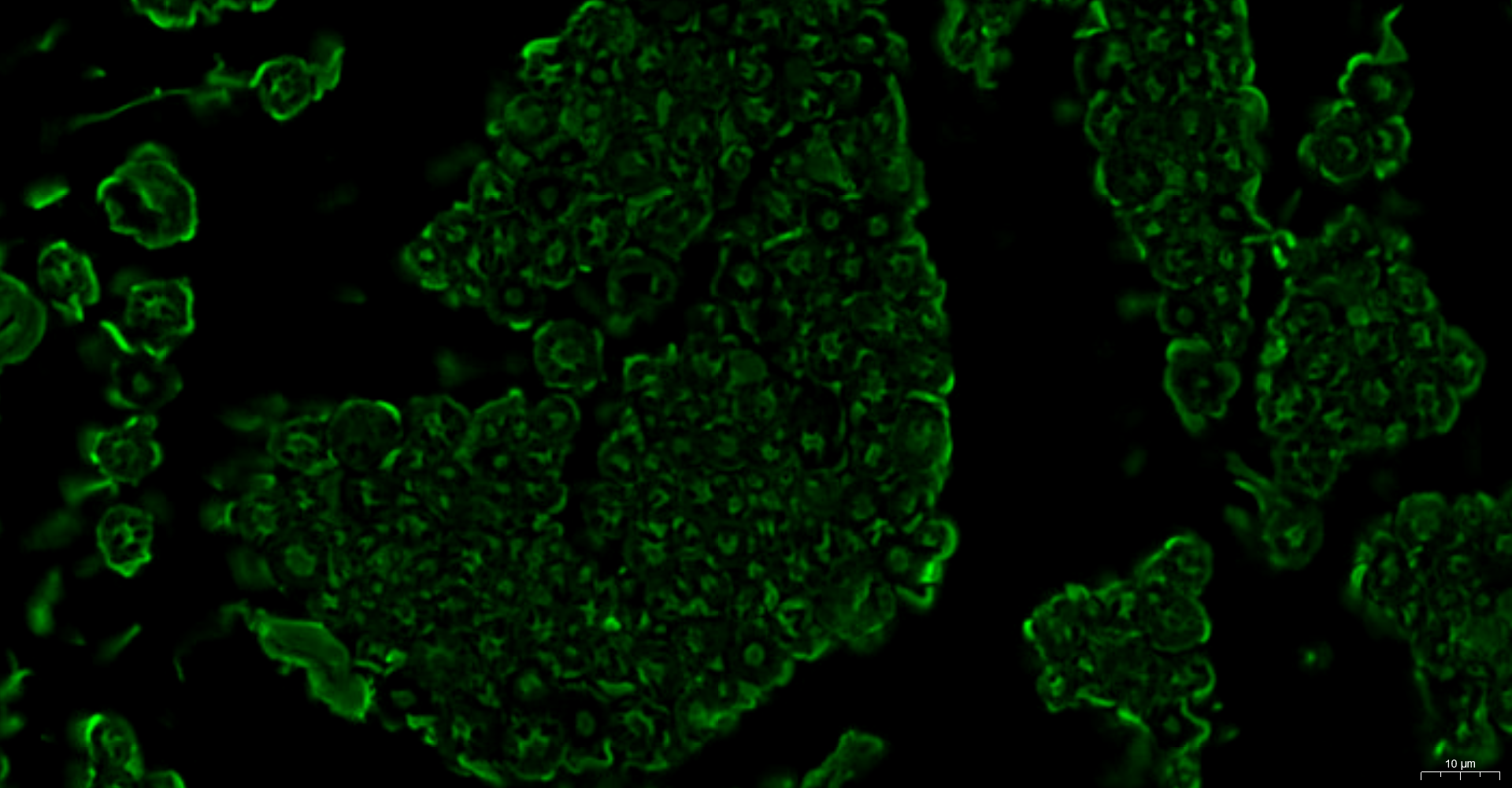

Supplement: Supplementary file 1 [file DataSheet3.ZIP › Immunofluorescence images∩╝êFor review purpose only∩╝ë/MA-/9MA(-) CYP1A2(488)100.0x.tif]

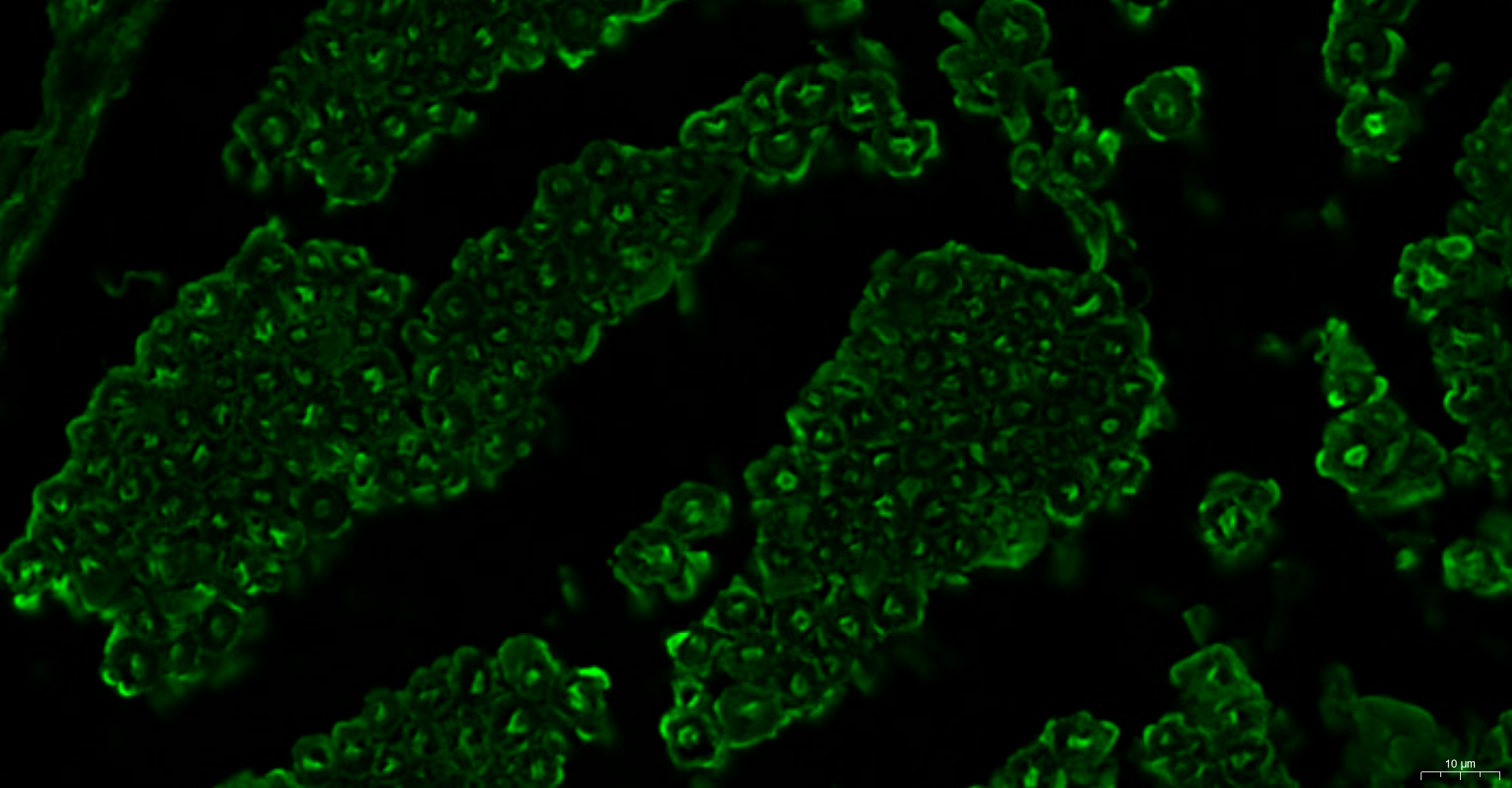

Supplement: Supplementary file 1 [file DataSheet3.ZIP › Immunofluorescence images∩╝êFor review purpose only∩╝ë/MA-/1MA(-)CYP1A2(488)100.0x.tif]

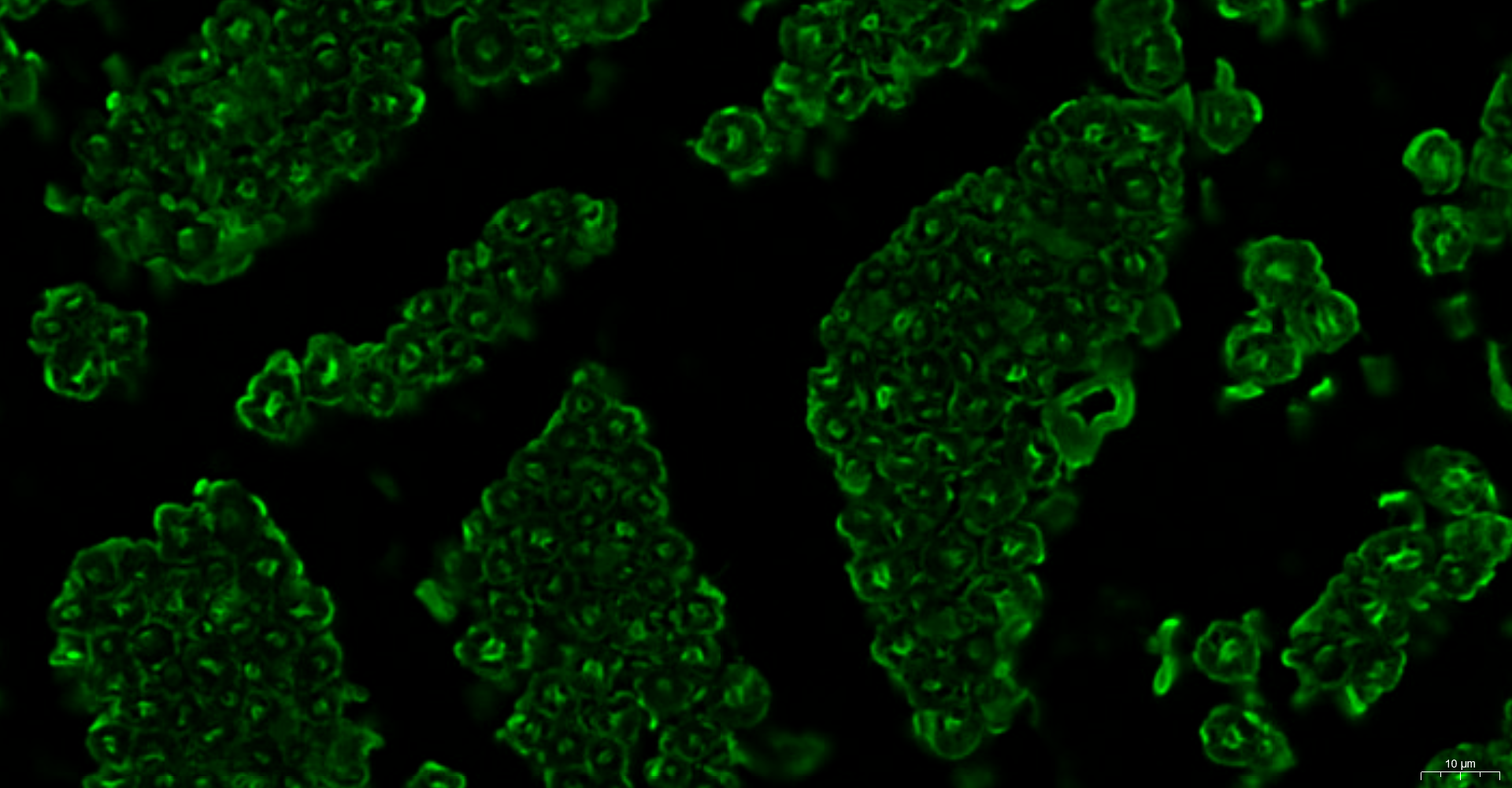

Supplement: Supplementary file 1 [file DataSheet3.ZIP › Immunofluorescence images∩╝êFor review purpose only∩╝ë/MA-/2MA(-) CYP1A2(488)100.0x.tif]

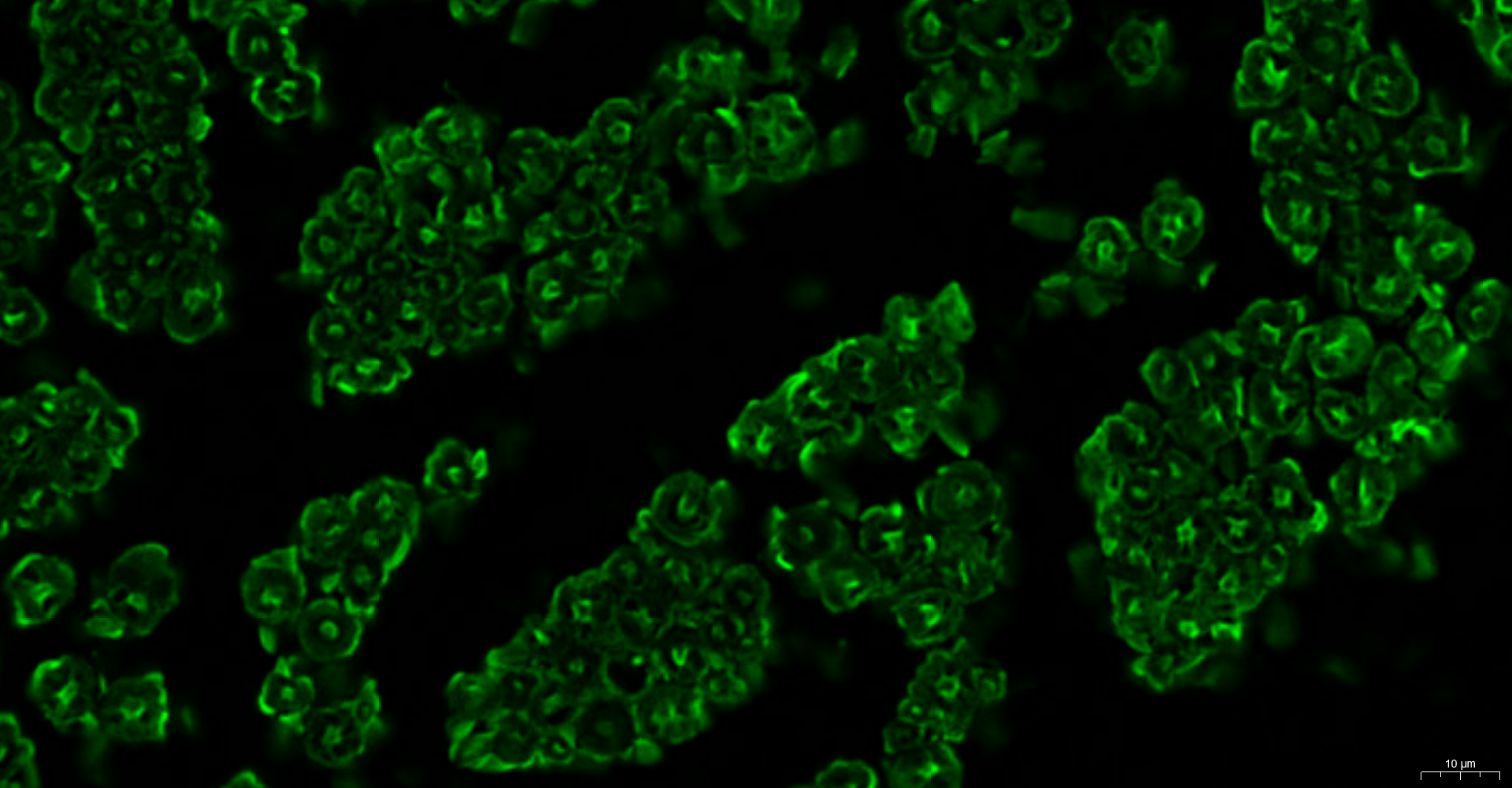

Supplement: Supplementary file 1 [file DataSheet3.ZIP › Immunofluorescence images∩╝êFor review purpose only∩╝ë/MA-/3MA(-)CYP1A2(488)100.0x.tif]

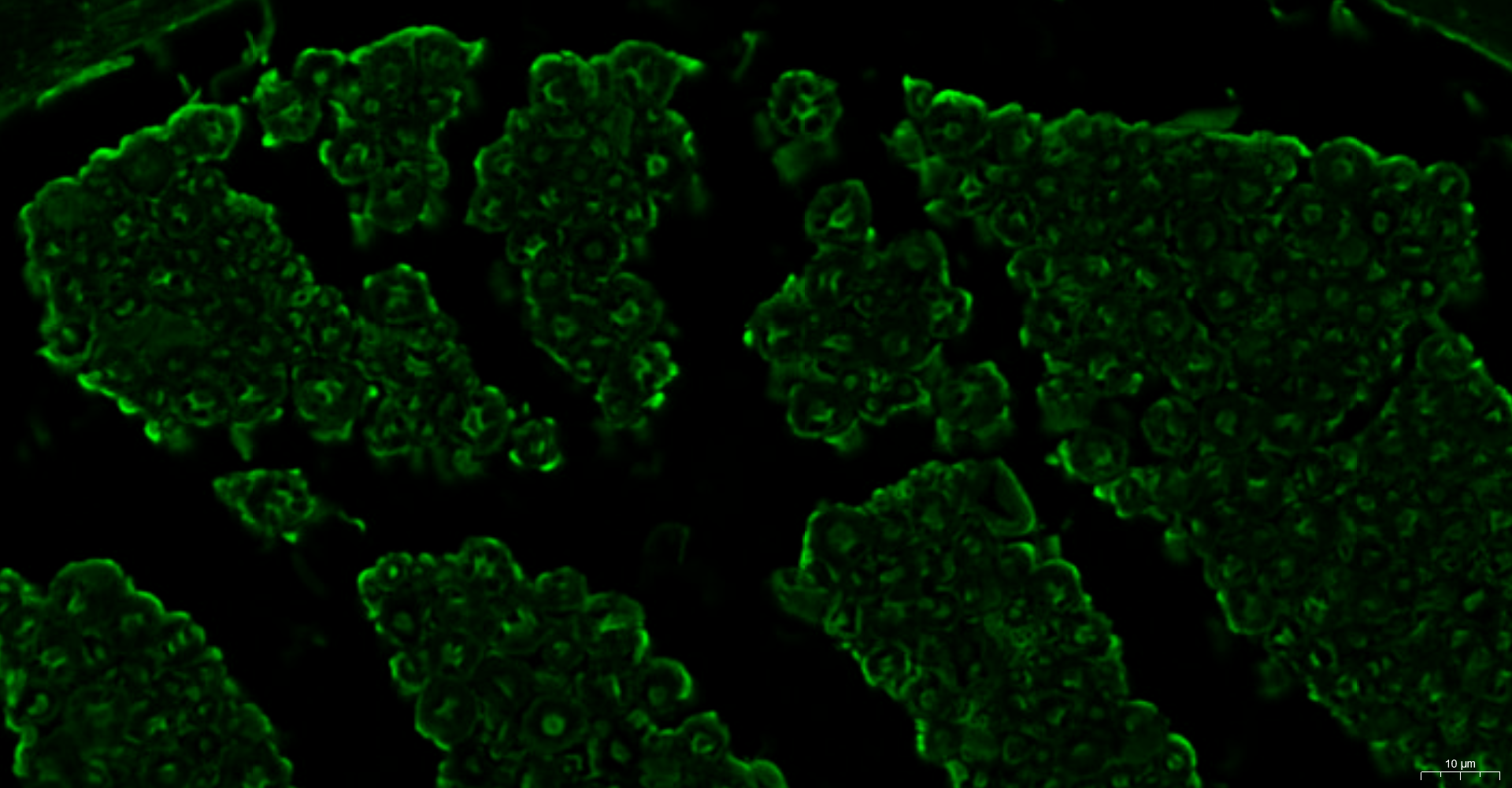

Supplement: Supplementary file 1 [file DataSheet3.ZIP › Immunofluorescence images∩╝êFor review purpose only∩╝ë/MA-/4MA(-)CYP1A2(488)100.0x.tif]
