# Supplementary material for: Multi-omics analysis revealed the role of CYP1A2 in the induction of mechanical allodynia in type 1 diabetes
Source: Front Genet. 2023 Mar 23;14:1151340. doi: 10.3389/fgene.2023.1151340 (PMC10076588; doi:10.3389/fgene.2023.1151340)
Supplement: Supplementary file 2 [file DataSheet1.PDF]

Supplementary Material

1 Supplementary Figures and Tables

1.1 Supplementary Figures 1

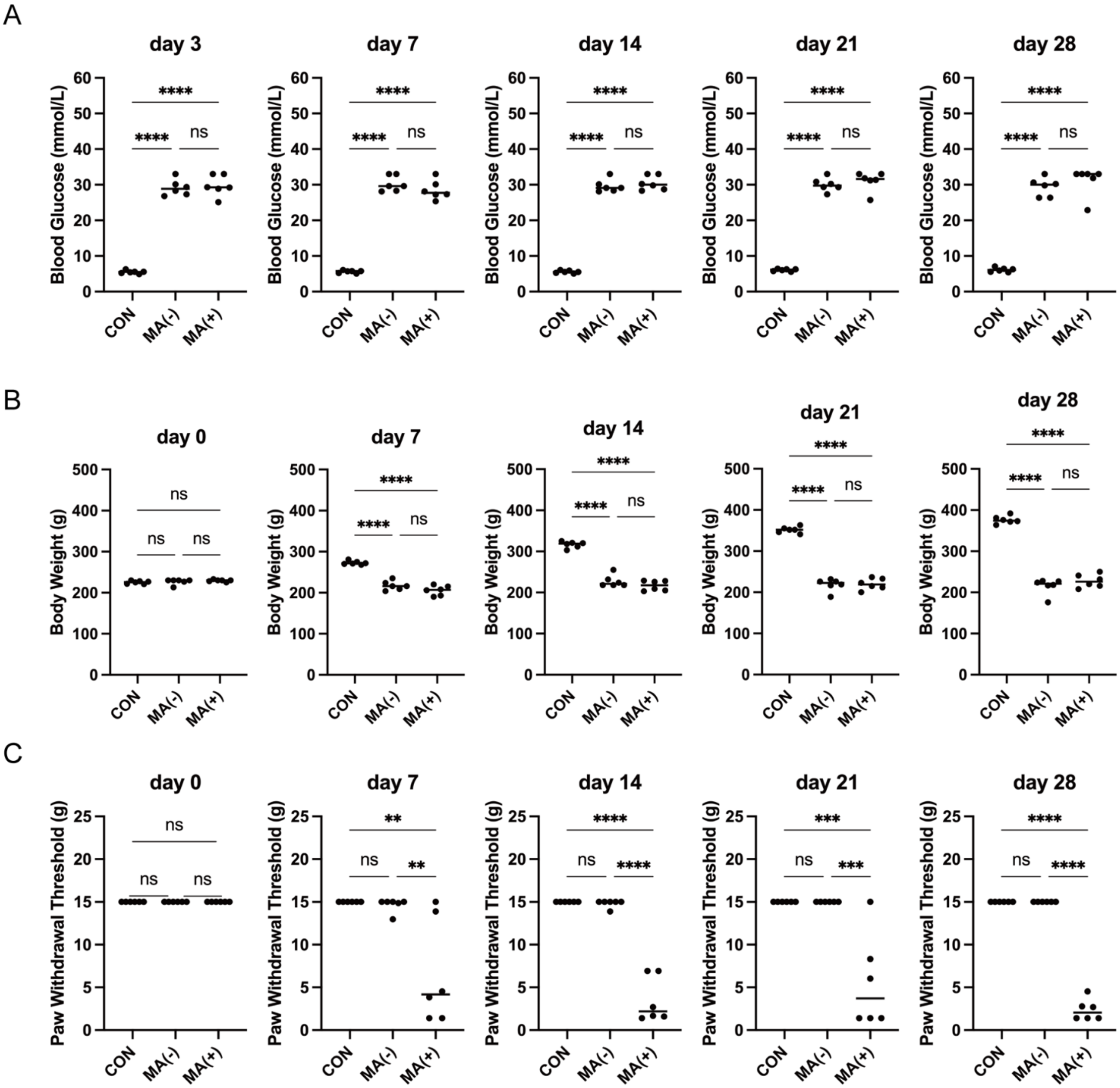

Supplementary Figure 1. The development of mechanical allodynia in diabetic rats.

A: Blood glucose levels of rats in different groups. (n = 6) B: Body weight of rats in different groups. C:Paw Withdrawal Threshold in different groups. *p＜0 .05; **p＜0.01; ***p＜0.001; ****p＜0.0001; ns, no significant.

1.2 Supplementary Figures 2

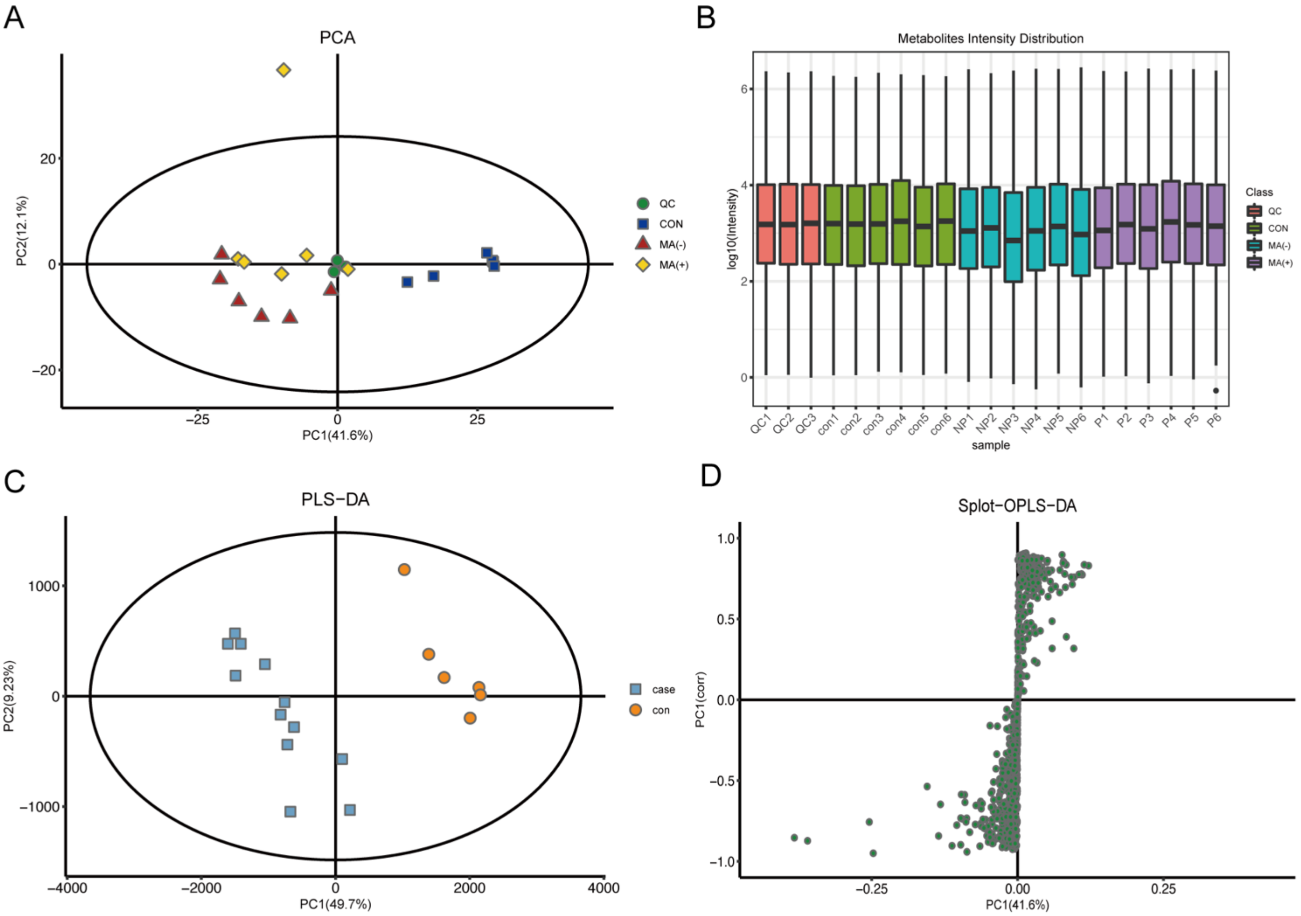

Supplementary Figure 2. Quality control and data analysis of lipidomics

A: Principle Component Analysis of all samples. B: Boxplot of Sample metabolite intensity. C: Explain and predict differences by Partial least squares-discriminant analysis. D: Splot, the metabolites closer to the upper right and lower left corners indicate their more significant differences. 6 rats in each group.

1.3 Supplementary Figures 3

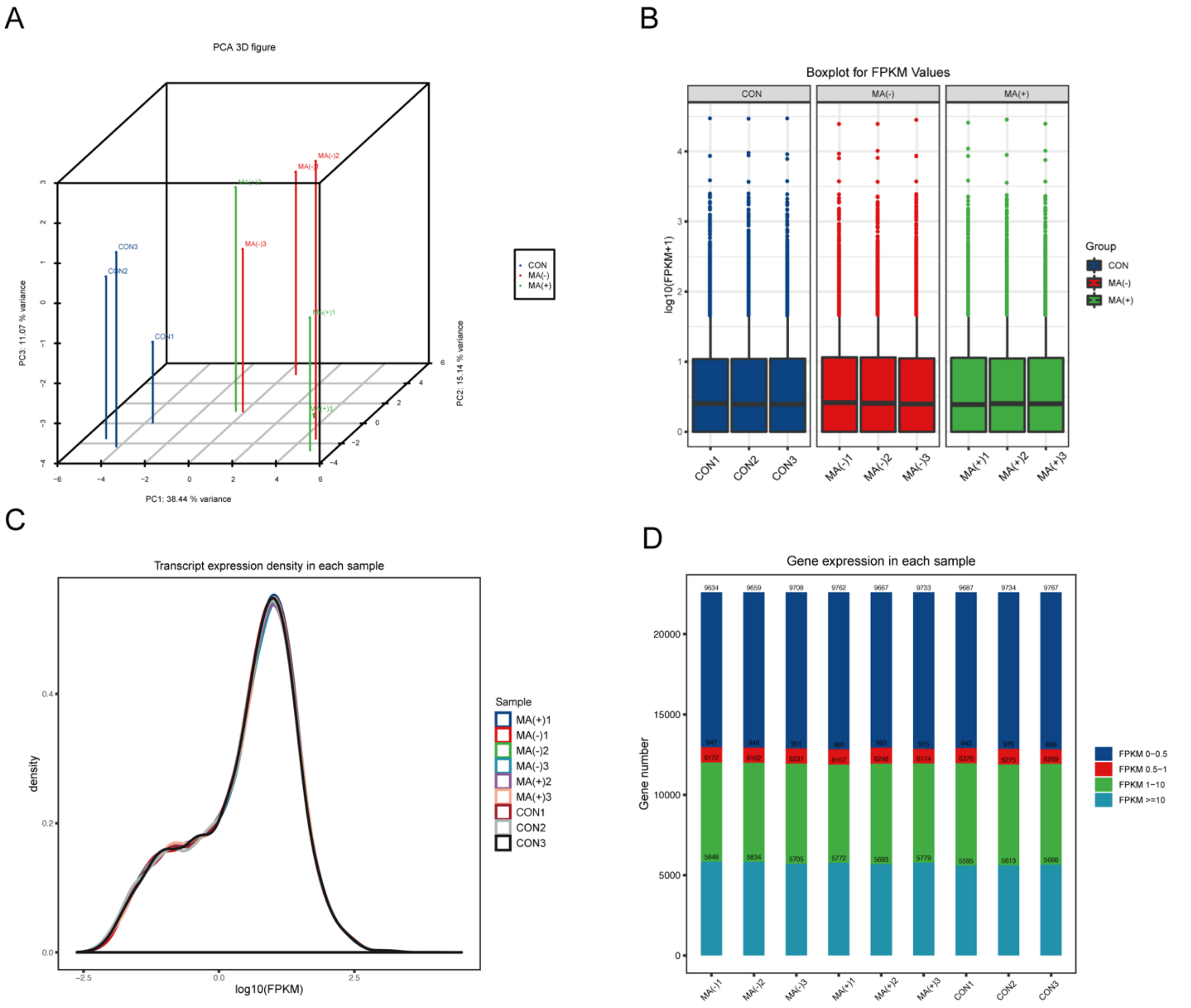

Supplementary Figure 3. Quality control and data analysis of transcriptomics

A: Principle Component Analysis of all samples. B: Box plot of FPKM values for each sample genes. C: Density distribution of FPKM values for each sample genes. D: Expression distribution of FPKM values for each sample genes. 6 rats in each group.

1.4 Supplementary Figures 4

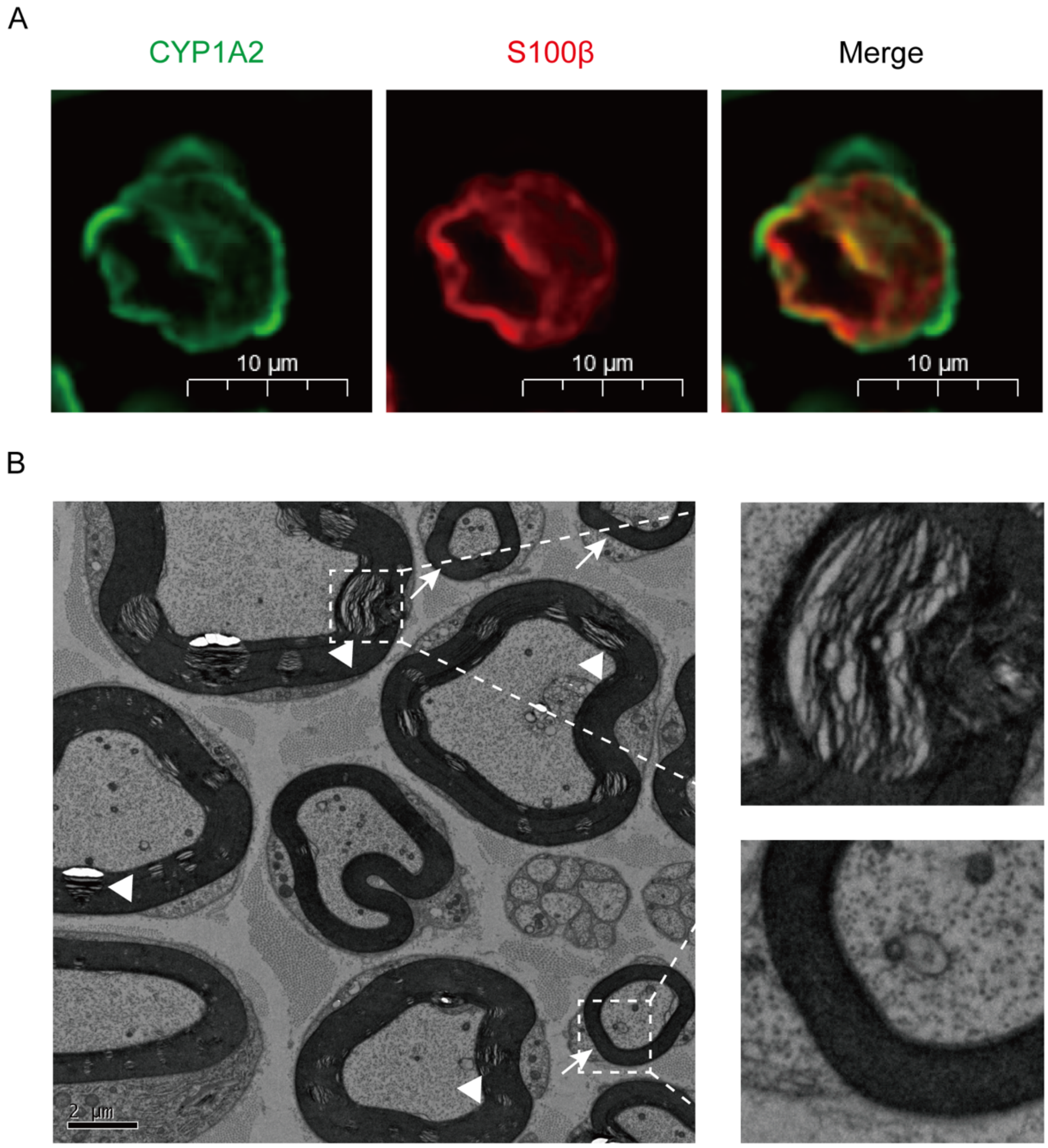

Supplementary Figure 4. Immunofluorescence staining and TEM scanning of sciatic nerve.

A: Immunofluorescence staining of the sciatic nerve for CYP1A2(Green) and S100β(Red).

B: TEM scanning of sciatic nerve of rat in MA(+) group.Myelin sheath degeneration and lipid droplet accumulation occur mainly in the thick myelin(white triangle) sheath and rarely accumulate in the thin myelin sheath(white arrow).
