# Supplementary material for: Multi-omics analysis revealed the role of CYP1A2 in the induction of mechanical allodynia in type 1 diabetes
Source: Front Genet. 2023 Mar 23;14:1151340. doi: 10.3389/fgene.2023.1151340 (PMC10076588; doi:10.3389/fgene.2023.1151340)
Supplement: Supplementary file 3 [file DataSheet2.ZIP › raw data and data sheets(For review purpose only)/Please read this first.docx]

(1)Lipidomic and integrated analysis：

R software (version 3.6.2) was used for statistical analyses.

multivariate statistical analyses (PCA, PLS-DA, etc.) were analyzed using the ropls:1.22.0 package of R software.

univariate statistical correlation was performed using the base package:stats:4.0.5.

mapping analysis packages included:pheatmap:1.0.12; ggplot3.3.6; ggrepel:0.9.1; dplyr:1.0.9.

corrplot:0.92 for correlation analysis.

(2)Transcriptomic analysis:

<https://github.com/fjxc1893/RNA-Seq> , Here is the generic code for quality control, comparison and quantification of RNA-seq; and a description of the version of R and related R packages (no specific code is provided for R).

(3) In the raw data of lipidomics and transcriptomics:

the con group is the CON group

the NP group is the MA(-) group

the P group is the MA(+) group.

All data sheets are For review purpose only.
